# Effect of Enteral Lipid Supplement on Severe Retinopathy of Prematurity: A Randomized Clinical Trial

**DOI:** 10.1001/jamapediatrics.2020.5653

**Published:** 2021-02-01

**Authors:** Ann Hellström, Anders K. Nilsson, Dirk Wackernagel, Aldina Pivodic, Mireille Vanpee, Ulrika Sjöbom, Gunnel Hellgren, Boubou Hallberg, Magnus Domellöf, Susanna Klevebro, William Hellström, Mats Andersson, Anna-My Lund, Chatarina Löfqvist, Anders Elfvin, Karin Sävman, Ingrid Hansen-Pupp, Anna-Lena Hård, Lois E. H. Smith, David Ley

**Affiliations:** 1 Department of Clinical Neuroscience, Institute of Neuroscience and Physiology, Sahlgrenska Academy, University of Gothenburg, Gothenburg, Sweden; 2 Department of Neonatology, Karolinska University Hospital and Institute, Astrid Lindgrens Children’s Hospital, Stockholm, Sweden; 3 Department of Women’s and Children’s Health, Karolinska Institutet and Karolinska Univeristy Hospital, Stockholm, Sweden; 4 Institute of Health Care Science, Sahlgrenska Academy, University of Gothenburg, Gothenburg, Sweden; 5 Institute of Biomedicine, Sahlgrenska Academy, University of Gothenburg, Gothenburg, Sweden; 6 Department of Pediatrics, Institution of Clinical Science Intervention and Technology (CLINTEC), Karolinska Institutet and Department of Neonatology, Karolinska University Hospital, Stockholm, Sweden; 7 Institute of Cinical Science, Department of Pediatrics, Umeå University Hospital, Umeå, Sweden; 8 Department of Clinical Science and Education, Stockholm South General Hospital, Karolinska Institutet, Sweden; 9 Institute of Clinical Sciences, Sahlgrenska Academy, Department of Pediatrics, University of Gothenburg, Gothenburg, Sweden; 10 Region Västra Götaland, Department of Neonatology, The Queen Silvia Children’s Hospital, Sahlgrenska University Hospital, Gothenburg, Sweden; 11 Department of Pediatrics, Institute of Clinical Sciences Lund, Lund University and Skane University Hospital, Lund, Sweden; 12 Department of Ophthalmology, Boston Children’s Hospital, Harvard Medical School, Boston, Massachusetts

## Abstract

**Importance:**

Lack of arachidonic acid (AA) and docosahexaenoic acid (DHA) after extremely preterm birth may contribute to preterm morbidity, including retinopathy of prematurity (ROP).

**Objective:**

To determine whether enteral supplementation with fatty acids from birth to 40 weeks’ postmenstrual age reduces ROP in extremely preterm infants.

**Design, Setting, and Participants:**

The Mega Donna Mega trial, a randomized clinical trial, was a multicenter study performed at 3 university hospitals in Sweden from December 15, 2016, to December 15, 2019. The screening pediatric ophthalmologists were masked to patient groupings. A total of 209 infants born at less than 28 weeks’ gestation were tested for eligibility, and 206 infants were included. Efficacy analyses were performed on as-randomized groups on the intention-to-treat population and on the per-protocol population using as-treated groups. Statistical analyses were performed from February to April 2020.

**Interventions:**

Infants received either supplementation with an enteral oil providing AA (100 mg/kg/d) and DHA (50 mg/kg/d) (AA:DHA group) or no supplementation within 3 days after birth until 40 weeks’ postmenstrual age.

**Main Outcomes and Measures:**

The primary outcome was severe ROP (stage 3 and/or type 1). The secondary outcomes were AA and DHA serum levels and rates of other complications of preterm birth.

**Results:**

A total of 101 infants (58 boys [57.4%]; mean [SD] gestational age, 25.5 [1.5] weeks) were included in the AA:DHA group, and 105 infants (59 boys [56.2%]; mean [SD] gestational age, 25.5 [1.4] weeks) were included in the control group. Treatment with AA and DHA reduced severe ROP compared with the standard of care (16 of 101 [15.8%] in the AA:DHA group vs 35 of 105 [33.3%] in the control group; adjusted relative risk, 0.50 [95% CI, 0.28-0.91]; *P* = .02). The AA:DHA group had significantly higher fractions of AA and DHA in serum phospholipids compared with controls (overall mean difference in AA:DHA group, 0.82 mol% [95% CI, 0.46-1.18 mol%]; *P* < .001; overall mean difference in control group, 0.13 mol% [95% CI, 0.01-0.24 mol%]; *P* = .03). There were no significant differences between the AA:DHA group and the control group in the rates of bronchopulmonary dysplasia (48 of 101 [47.5%] vs 48 of 105 [45.7%]) and of any grade of intraventricular hemorrhage (43 of 101 [42.6%] vs 42 of 105 [40.0%]). In the AA:DHA group and control group, respectively, sepsis occurred in 42 of 101 infants (41.6%) and 53 of 105 infants (50.5%), serious adverse events occurred in 26 of 101 infants (25.7%) and 26 of 105 infants (24.8%), and 16 of 101 infants (15.8%) and 13 of 106 infants (12.3%) died.

**Conclusions and Relevance:**

This study found that, compared with standard of care, enteral AA:DHA supplementation lowered the risk of severe ROP by 50% and showed overall higher serum levels of both AA and DHA. Enteral lipid supplementation with AA:DHA is a novel preventive strategy to decrease severe ROP in extremely preterm infants.

**Trial Registration:**

ClinicalTrials.gov Identifier: NCT03201588

## Key Points

**Question:**

Does enteral fatty acid supplementation with arachidonic acid (AA) and docosahexaenoic acid (DHA) from birth to 40 weeks’ postmenstrual age reduce severe retinopathy of prematurity (ROP) in extremely preterm infants?

**Findings:**

This randomized clinical trial found that enteral AA and DHA supplementation lowered the risk of severe ROP by 50%. In addition, the group that received enteral AA and DHA supplementation showed higher serum levels of both AA and DHA compared with controls.

**Meaning:**

Supplementing the diet of the most immature infants born at less than 27 weeks’ gestational age with an enteral lipid solution with AA:DHA had no significant adverse effects and seems to be a promising intervention to prevent sight-threatening ROP and thereby reduce visual impartment and blindness.

## Teaser

This randomized clinical trial examines whether enteral supplementation with arachidonic acid and docosahexaenoic acid from birth to 40 weeks’ postmenstrual age reduces retinopathy of prematurity in extremely preterm infants.

## Introduction

Extremely preterm infants have a high incidence of neonatal morbidities, including retinopathy of prematurity (ROP), a neurovascular disease with initial suppression of retinal blood vessel growth followed by pathologic neovascularization that can cause blindness.^1^ Treatment of severe ROP aims to cause regression of pathologic neovascularization and prevent retinal detachment by reducing the action of vascular endothelial growth factor (VEGF). Laser therapy is performed under general anesthesia and destroys the hypoxic VEGF-producing peripheral retina. Anti-VEGF therapy comprises intraocular injections, often repeated, of antibodies against VEGF. These substances enter the circulation, and concerns have been raised about their effects on growing tissues. Retinopathy of prematurity correlates with reduced brain volumes and poor psychomotor development.^2^

Infants born extremely preterm miss the third-trimester transfer from the mother of the ω-6 long chain polyunsaturated fatty acid (LCPUFA) arachidonic acid (AA) and the ω-3 LCPUFA docosahexaenoic acid (DHA). Arachidonic acid and DHA are critical constituents of the retina and the brain, and deficiencies are associated with vascular complications of preterm birth.^3,4^ The AA lipid fraction is 2-fold higher in fetal blood than maternal blood throughout gestation, while the DHA lipid fraction is similar to the maternal fraction until approximately 30 weeks’ gestational age (GA), then increases concomitant with rapid brain growth.^5^ The lipid emulsions currently used in parenteral nutrition contain insufficient or no AA and DHA. Breast milk does not provide enough AA and DHA to fulfill the early requirements of extremely preterm infants.^6,7^ Thus, extremely preterm infants accumulate AA and DHA deficits during hospitalization.^8,9^

Several studies suggest that low ω-3 LCPUFAs are associated with ROP risk. Increased intake of ω-3 protected against pathologic neovascularization in mouse oxygen-induced retinopathy.^10,11^ The DHA oxidation product 4-hydroxy-docosahexaenoic acid inhibited pathologic neovascularization independently of its anti-inflammatory effects.^12^ Decreased frequency of any ROP or treatment for ROP was reported with the use of fish oil–based parenteral lipid solutions rich in ω-3.^13,14,15^ However, parenteral nutrition with 15% fish oil vs an olive oil–based lipid solution substantially reduced AA serum levels and AA to DHA ratios.^16^ A lower AA serum fraction was associated with increased frequency of severe ROP.^17^

A few studies have been performed using enteral DHA supplementation given early after birth to preterm infants. A reduction in stage 3 ROP was found in infants with a birth weight of 1000 to 1500 g who received enteral DHA for 14 days.^18^ Collins et al^19^ reported no benefit and possibly increased risk of bronchopulmonary dysplasia (BPD) with DHA supplementation from the first enteral feeding to 36 weeks’ postmenstrual age (PMA) or hospital discharge in 1273 infants with a GA less than 29 weeks; no effect on ROP was reported.

The primary aim of this trial was to study the frequency of severe (stage 3 and/or type 1) ROP with or without AA and DHA supplementation. Secondary aims were to investigate the effects of AA and DHA supplementation on serum phospholipid fatty acid composition and the rates of other complications of prematurity. This study adds to current knowledge by studying the effects of an enteral lipid supplement containing both AA and DHA given to extremely preterm infants stratified into GA-based groups.

## Methods

### Study Design

The Mega Donna Mega trial was a randomized clinical trial performed at 3 centers in Sweden from December 15, 2016, to December 15, 2019. Regionala etikprövningsnämnden in Göteborg approved the trial protocol (trial protocol in Supplement 1). Parents or guardians provided written informed consent. An independent data safety committee reviewed trial safety twice with access to key outcomes. This study followed the Consolidated Standards of Reporting Trials (CONSORT) reporting guideline.

### Treatment Groups

Infants born before 28 weeks’ GA were eligible to participate. Exclusions were based on specific criteria (eAppendix 1 in Supplement 2). The target range for oxygen saturation as measured by pulse oximetery was from 91% to 95% during oxygen supplementation.

### Randomization and Masking

Neonatologists and/or research nurses assessed infants for eligibility. Those who met eligibility criteria and had written informed consent from parents or guardians were randomly assigned to a computer-generated schedule that used balanced variable blocks within each site in a ratio of 1:1. Randomization was stratified according to GA of less than 25 weeks (planned 84 infants), 25 to 26 weeks (planned 84 infants), or 27 weeks (planned 42 infants) as well as according to center. Multiple births were randomized to the same group. The local principal investigator at each site (eg, the neonatologist) generated the random allocation sequence, enrolled participants, and assigned participants to interventions. Ophthalmologists, unaware of randomization assignments, screened for ROP according to national guidelines.

### Trial Intervention

The AA and DHA 2:1 supplement (Formulaid, DSM Nutritional Products Inc) comprises a triglyceride oil containing AA from fungi and DHA from algae. The emulsion (0.39 mL/kg/d) was administered enterally from within 72 hours after birth to 40 weeks’ PMA. Infants in the AA:DHA group received 100 mg of AA plus 50 mg of DHA per kg/d. The supplemented dose corresponded to estimated fetal accretion.^20,21^ For dose adjustments, see eAppendix 2 in Supplement 2. Apart from the administration of the trial emulsion, neonatologists directed nutritional management according to national guidelines (eAppendix 2 in Supplement 2).

### Primary Outcome

There was no change to trial outcomes after the trial commenced. The primary outcome was the incidence of severe ROP (ie, ROP stage 3 and/or type 1 ROP) according to the international ROP classification.^22^ Treatment of ROP was undertaken according to international standards.^23^

### Secondary Outcomes

Secondary outcomes included levels of serum phospholipid fatty acids, BPD, intraventricular hemorrhage (IVH), patent ductus arteriosus (PDA), necrotizing enterocolitis, measures of safety and tolerance, death, and growth measured up to 40 weeks’ PMA (eAppendix 1 in Supplement 2). A whole-blood specimen (0.6 mL) for serum phospholipid fatty acid analysis was obtained at birth and at postnatal days 3, 7, and 14 and thereafter biweekly until postmenstrual week 29 and at 30, 32, 34, 36, and 40 weeks’ PMA.^16^ Methods for lipid extraction and fatty acid analysis can be found in eAppendix 4 in the Supplement.

### Statistical Analysis

With an estimated incidence of severe ROP among infants born at less than 28 weeks’ GA of 42%,^24^ we calculated that 210 infants should be enrolled to compensate for protocol violations and dropouts (eg, a 15% death rate) for 80% power to detect a 50% relative difference between trial groups in the incidence of severe ROP, at a 2-tailed α level of 0.05. Efficacy analyses were performed on as-randomized groups on the intention-to-treat (ITT) population and on the per-protocol (PP) population using as-treated groups, according to a prespecified statistical analysis plan (Supplement 1). The PP population included infants who had more than 70% adherence to AA:DHA doses and a finalized ROP stage. The safety population included all enrolled infants.

Primary analysis was performed using Poisson regression, adjusted for GA and center, describing event rates, relative risks, and 95% CIs. Cumulative incidence functions of severe ROP were described using the Kaplan-Meier technique. Early dropouts and deaths before the final evaluation of maximum ROP were considered as nonevents and were censored at the time of early discontinuation in the primary analysis. In the sensitivity analysis, death before final evaluation of maximum ROP was handled as a competing risk. The difference between the groups was described by a subdistribution hazard ratio with 95% CIs using the Fine-Gray method and was tested with the Gray test.

Analyses of BPD and necrotizing enterocolitis were performed using Poisson regression, analyses of IVH grades were performed using the Mantel-Haenszel χ^2^ test, and analyses of PDA were performed using the Fisher exact test. Longitudinal postnatal LCPUFAs (in units of mole percent [mol%]) AA and DHA and longitudinal growth data were analyzed using mixed models for repeated measures, applying the lowest Akaike Information Criterion when selecting covariance structure. The overall analyses included treatment group, visit, interaction between treatment group and visit, and the corresponding LCPUFA at birth as fixed effects and LCPUFA after birth as a dependent variable, handling visit as a repeated measure with unstructured covariance pattern estimated per treatment group. The diagnostic plots were visually reviewed, and the method assumptions were found to be satisfied.

Confirmatory analyses were prespecified for the ITT population according to the fixed sequential testing and inheriting a significance mass of 0.05 to the next variable in order: (1) severe ROP, (2 and 3) overall levels of AA and DHA (mol%), (4) BPD, and (5) IVH grade. Time to death was described by Kaplan-Meier graphs, tested with a log-rank test, and described by hazard ratios and 95% CI using Cox proportional hazards regression. Outcomes and time to death were described by GA randomization strata. Analyses were performed using SAS software, version 9.4 (SAS Institute Inc). *P* < .05 was considered significant.

## Results

### Trial Participants

**Figure 1.**
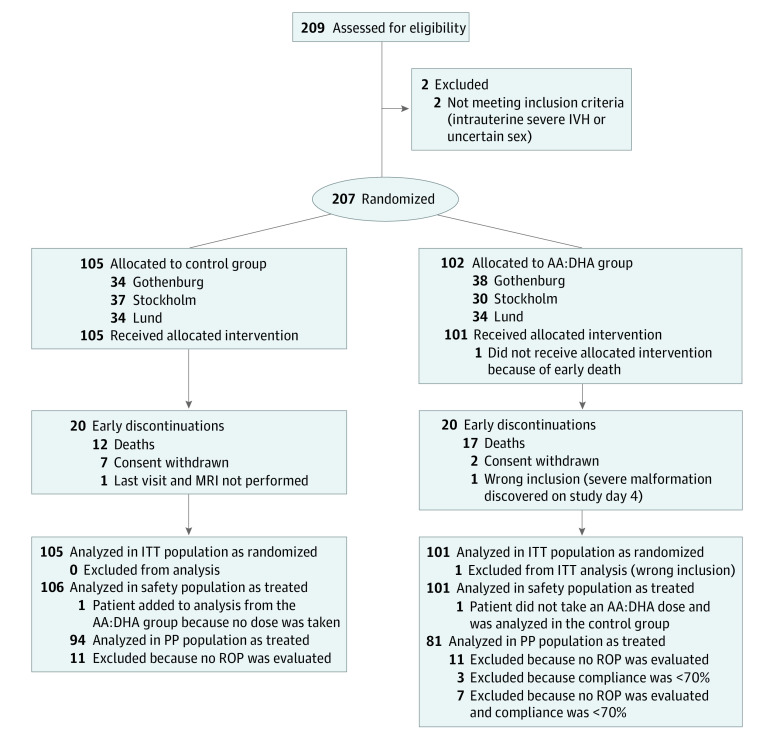
Flow Diagram of 209 Eligible Infants for Inclusion in the Arachidonic Acid (AA) and Docosahexaenoic Acid (DHA) Enteral Supplementation Randomized Multicenter Trial ITT indicates intention-to-treat; IVH, intraventricular hemorrhage; MRI, magnetic resonance imaging; PP, per-protocol; and ROP, retinopathy of prematurity.

From December 15, 2016, to August 7, 2019, a total of 209 infants were tested for eligibility, and 207 were included in the trial (Figure 1). The study was ended when 210 infants had been entered into the electronic clinical record form system; 1 patient was later found to be a virtual test patient. The baseline characteristics of the infants and mothers were similar in the 2 treatment groups (Table 1^25^; eTable 1 in Supplement 2). In the ITT population, among the 105 infants in the control group, the mean (SD) GA was 25.5 (1.4) weeks, and 46 infants (43.8%) were female. Among the 101 infants receiving AA and DHA, the mean (SD) GA was 25.5 (1.5) weeks, 43 infants (42.6%) were female, and the median duration of dosing with AA:DHA was 13.1 weeks (range, 0.0-17.4 weeks). A mean (SD) of 88.9% (20.5%) of ordered doses (interquartile range, 86.1%-100%) were given; 10 infants received less than 70% of planned doses. The mean (SD) baseline fraction of AA and DHA in serum did not differ between the control group and the AA:DHA group (AA, 12.6 [2.4] mol% vs 12.6 [2.6] mol%; DHA, 2.28 [0.68] mol% vs 2.37 [0.82] mol%) (Table 1).^25^ During the first postnatal month, there was no difference in macronutrient intake between the groups (eTable 2 in Supplement 2).

**Table 1.** Baseline Characteristics (Intention-to-Treat Population)

Characteristic	Randomized group, No. (%)	
	Control (n = 105)	AA:DHA (n = 101)
Gestational age, wk		
Mean (SD)	25.5 (1.4)	25.5 (1.5)
Median (range)	25.6 (22.9 to 27.9)	25.4 (22.3 to 27.9)
No.	105	101
Gestational age at birth, wk		
<25	39 (37.1)	37 (36.6)
25-26	47 (44.8)	41 (40.6)
≥27	19 (18.1)	23 (22.8)
Female	46 (43.8)	43 (42.6)
Birth weight, g		
Mean (SD)	777 (197)	797 (197)
Median (range)	758 (411 to 1330)	775 (455 to 1345)
No.	105	101
Birth weight, SDS		
Mean (SD)	−0.848 (1.203)	−0.652 (1.227)
Median (range)	−0.536 (−4.083 to 1.222)	−0.351 (−5.058 to 1.744)
No.	105	101
Birth length, cm		
Mean (SD)	32.8 (2.7)	32.9 (2.6)
Median (range)	32.7 (27.4 to 41.0)	33.0 (27.0 to 39.0)
No.	86	86
Birth length, SDS		
Mean (SD)	−1.30 (1.53)	−1.26 (1.58)
Median (range)	−1.05 (−4.70 to 2.89)	−1.09 (−6.76 to 1.42)
No.	86	86
Birth head circumference, cm		
Mean (SD)	23.2 (1.8)	23.2 (1.9)
Median (range)	23.2 (19.7 to 27.5)	23.0 (18.7 to 27.5)
No.	102	98
Birth head circumference, SDS		
Mean (SD)	−0.654 (0.779)	−0.662 (0.782)
Median (range)	−0.630 (−2.499 to 1.196)	−0.586 (−2.860 to 0.845)
No.	102	98
Baseline AA in serum, mol%		
Mean (SD)	12.6 (2.4)	12.6 (2.6)
Median (range)	12.3 (7.4 to 17.5)	12.4 (8.0 to 19.9)
No.	101	99
Baseline DHA in serum, mol%		
Mean (SD)	2.28 (0.68)	2.37 (0.82)
Median (range)	2.28 (0.77 to 4.39)	2.17 (1.09 to 4.78)
No.	101	99
Plurality		
Single	81 (77.1)	84 (83.2)
Twin	21 (20.0)	14 (13.9)
Triplet	3 (2.9)	3 (3.0)
Center		
Gothenburg	34 (32.4)	38 (37.6)
Stockholm	37 (35.2)	29 (28.7)
Lund	34 (32.4)	34 (33.7)
Mother’s age, y		
Mean (SD)	31.9 (5.0)	32.0 (5.6)
Median (range)	31.1 (20.6 to 46.2)	32.2 (16.2 to 43.8)
No.	99	99
Parity (No. of children)		
Mean (SD)	1.70 (0.94)	1.73 (1.09)
Median (range)	1.00 (1.00 to 5.00)	1.00 (1.00 to 8.00)
No.	103	97
Cesarean delivery	65 (61.9)	61 (60.4)
Mothers		
With any medical history	37 (35.2)	48 (47.5)
With type 1 diabetes	0	2 (2.0)
With hypertension	14 (13.3)	10 (9.9)

Abbreviations: AA, arachidonic acid; DHA, docosahexaenoic acid; SDS, standard deviation score using method from Niklasson and Albertsson-Wikland.^25^

The safety population included 207 infants, and the PP population included 175 infants. Primary outcome data were available for 178 infants (86.0%); 29 infants (14.0%), 1 with type 1 ROP, died before 40 weeks’ PMA.

### Primary Outcome

**Figure 2.**
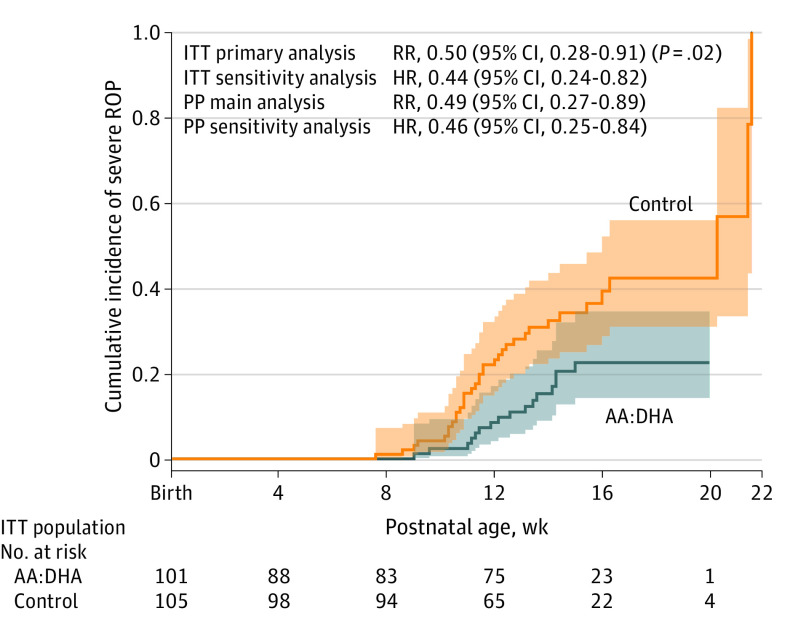
Cumulative Incidence Functions for Severe Retinopathy of Prematurity (ROP) (Intention-to-Treat [ITT] Population) Relative risks (RRs) with 95% CI from Poisson regression and subdistribution hazard ratios (HRs) with 95% CI from the survival analyses handling death as a competing risk, for the ITT and per-protocol (PP) population are presented. AA indicates arachidonic acid; and DHA, docosahexaenoic acid.

Severe ROP was reduced by 50% with AA and DHA supplementation vs no supplementation (16 of 101 infants [15.8%] in the AA:DHA group and 35 of 105 infants [33.3%] in the control group) (Figure 2 and Table 2), confirming the primary hypothesis with an adjusted relative risk of 0.50 (95% CI, 0.28-0.91; *P* = .02). In the sensitivity analysis, the hazard ratio was 0.44 (95% CI, 0.24-0.82; *P* = .006) (eTable 3 in Supplement 2). Similar results were shown for the PP population (eTable 4 in Supplement 2).

**Table 2.** Primary and Secondary Efficacy Analyses (Intention-to-Treat Population)

Outcome	Randomized group, No. (%)		Comparison: AA:DHA vs control (95% CI)	*P* value
	Control (n = 105)	AA:DHA (n = 101)		
**Primary outcomes**				
Severe ROP				
No. (%)	35 (33.3)	16 (15.8)	RR, 0.50 (0.28 to 0.91)	.02
Event rate (95% CI)^a^	13.1 (6.4 to 26.7)	6.6 (3.0 to 14.5)		
ROP stage^b^				
No ROP	48 (45.7)	52 (51.5)	NA	NA
ROP stages 1-2	22 (21.0)	33 (32.7)		
ROP stage 3 or type 1	35 (33.3)	16 (15.8)		
**Secondary outcomes**				
Overall postnatal molar ratio, LS means (95% CI)^c^				
AA	7.66 (7.42 to 7.90)	8.48 (8.20 to 8.76)	Difference in LS means, 0.82 (0.46 to 1.18)	<.001
DHA	2.07 (2.00 to 2.15)	2.20 (2.11 to 2.29)	Difference in LS means, 0.13 (0.01 to 0.24)	.03
Bronchopulmonary dysplasia				
No. (%)	48 (45.7)	48 (47.5)	RR, 1.11 (0.74 to 1.65)	.61
Event rate (95% CI)^a^	46.5 (35.0 to 61.7)	51.6 (38.9 to 68.4)		
Intraventricular hemorrhage				
No intraventricular hemorrhage	63 (60.0)	58 (57.4)	NA	NA
Grade I	11 (10.5)	16 (15.8)		
Grade II	14 (13.3)	17 (16.8)		
Grade III	5 (4.8)	6 (5.9)		
Grade IV	12 (11.4)	4 (4.0)		
Patent ductus arteriosus	51 (48.6)	53 (52.5)	NA	NA
Necrotizing enterocolitis				
No. (%)	11 (10.5)	10 (9.9)	RR, 0.96 (0.41 to 2.26)	NA
Event rate (95% CI)^a^	8.1 (4.5 to 14.6)	7.7 (4.2 to 14.4)		
Overall weight, LS means (95% CI), SDS^c^	−1.65 (−1.79 to −1.51)	−1.79 (−1.96 to −1.62)	Difference in LS means, 0.14 (−0.07 to 0.36)	NA
Overall length, LS means (95% CI), SDS^c^	−2.93 (−3.17 to −2.69)	−3.05 (−3.32 to −2.78)	Difference in LS means, 0.12 (−0.23 to 0.48)	NA
Overall head circumference, LS means (95% CI), SDS^c^	−1.47 (−1.63 to −1.32)	−1.49 (−1.66 to −1.33)	Difference in LS means, 0.02 (−0.21 to 0.25)	NA

Abbreviations: AA, arachidonic acid; DHA, docosahexaenoic acid; LS, least-squares; NA, not applicable; ROP, retinopathy of prematurity; RR, relative risk; SDS, standard deviation score computed using method from Niklasson and Albertsson-Wikland.^25^

^a^ Per 1000 person-weeks.

^b^ Descriptive only, not part of the planned analyses.

^c^ Overall effect was analyzed using mixed models for repeated measures including treatment group, visit, interaction between treatment group and visit, and at birth value as fixed effects. Unstructured covariance pattern was applied by treatment group.

The numerically largest reduction in severe ROP was observed among infants with GA less than 25 weeks (relative risk, 0.42 [95% CI, 0.19-0.92]). The corresponding relative risk was 0.69 (95% CI, 0.27-1.76) for infants of 25 to 26 weeks’ GA. Of infants born at 27 weeks’ GA, 1 in the control group developed severe ROP (eTable 5 in Supplement 2).

### Secondary Outcomes

#### AA and DHA Levels

**Figure 3.**
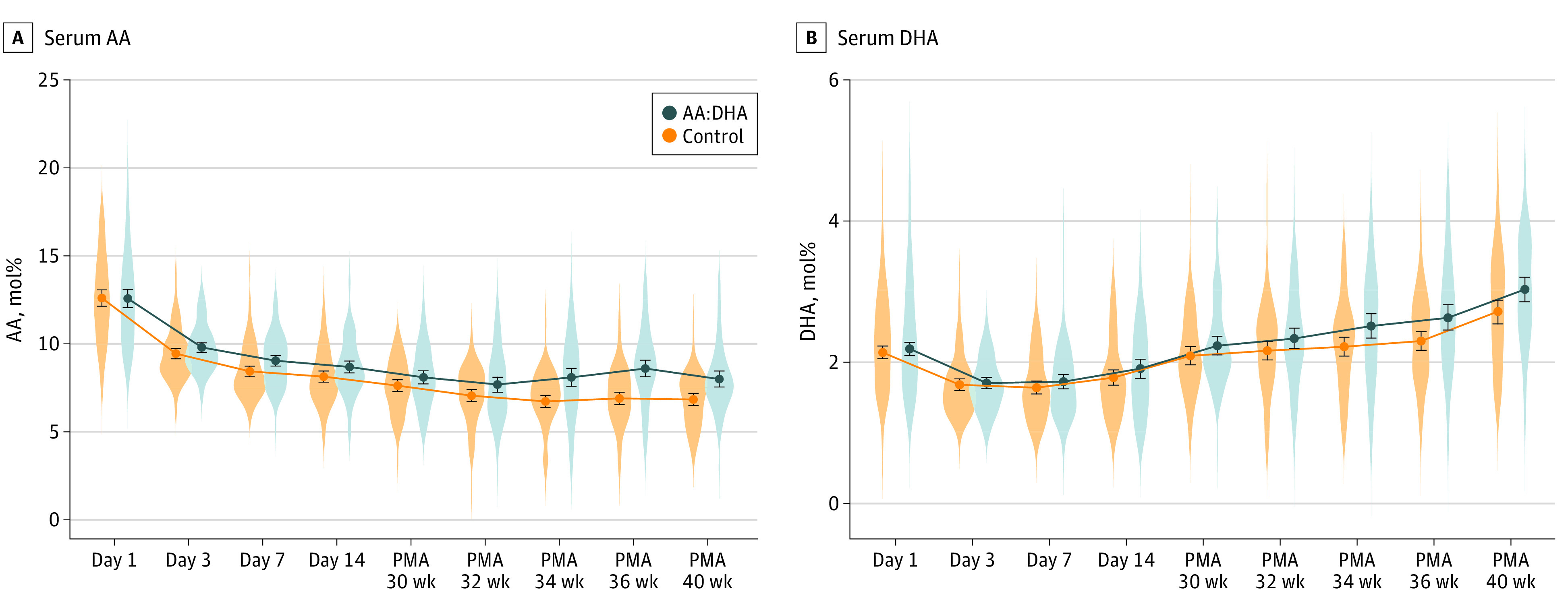
Longitudinal Postnatal Molar Ratio of Serum Arachidonic Acid (AA) and Docosahexaenoic Acid (DHA) of Total Fatty Acids in the AA:DHA and Control Groups (Intent-to-Treat Population) PMA indicates postmenstrual age.

The fraction of serum AA was significantly higher in the AA:DHA group than in the control group, with an overall mean difference from birth to 40 weeks’ PMA of 0.82 mol% (95% CI, 0.46-1.18 mol%; *P* < .001) (Table 2; eTable 6 in Supplement 2). Numerically higher mean AA levels in the AA:DHA group compared with the control group were observed throughout the follow-up period (Figure 3A). The overall mean difference for DHA between the AA:DHA group and the control group was 0.13 mol% (95% CI, 0.01-0.24 mol%; *P* = .03) (Table 2). Numerically higher mean DHA levels could be seen from PMA 34 weeks and onward (Figure 3B). Analyses performed for the PP population showed similar results (eTable 4 and eTable 7 in Supplement 2). The numerically highest increase in AA and DHA serum levels were seen in the most immature infants, and no differences were observed between infants in the AA:DHA group and infants in the control group who were born at 27 weeks’ GA (eTable 5 and eFigure 1A, B, and C in Supplement 2).

### Morbidities

The rate of BPD in the ITT analysis was 48 of 101 (47.5%) in the AA:DHA group and 48 of 105 (45.7%) in the control group (relative risk, 1.11 [95% CI, 0.74-1.65; *P* = .61; Table 2). This analysis ended the fixed sequential testing for the confirmatory analyses.

There were no significant differences in major clinical outcomes (necrotizing enterocolitis, PDA, IVH, or postnatal growth) between the groups in the ITT analysis (Table 2) or in the sensitivity or PP analyses (eTable 3 and eTable 4 in Supplement 2). Analyzing the 3 GA strata separately, we found similar proportions of BPD and PDA in the AA:DHA and control groups in the 2 lowest GA strata but numerically higher frequencies of BPD and PDA in infants born at 27 weeks’ GA in the AA:DHA group (BPD, 10 of 23 vs 4 of 19; PDA, 9 of 23 vs 2 of 19) (eTable 5 in Supplement 2).

### Safety

Nearly all infants experienced at least 1 adverse event (AA:DHA group, 99 [98.0%]; control group, 105 of 106 [99.1%]) (eTable 8, eTable 9, and eTable 10 in Supplement 2). There were similar numbers of serious adverse events in both groups: 26 patients in the AA:DHA group (25.7%) and 26 patients in the control group (24.8%) experienced 1 or more SAE. A total of 42 infants in the AA:DHA group (41.6%) and 53 infants in the control group (50.5%) developed sepsis.

There were 29 of 207 deaths (14.0%) in the entire study population, with no significant between-group differences before 40 weeks’ PMA in the safety population: 16 of 101 (15.8%) in the AA:DHA group and 13 of 106 (12.3%) in the control group (hazard ratio, 1.32; 95% CI, 0.63-2.74; *P* = .46) (eFigure 2 in Supplement 2). Of the 16 deceased infants in the AA:DHA group, 5 had received a maximum 3 doses of the investigational product (eTable 11 in Supplement 2). The 2 deceased infants born at 27 weeks’ GA were part of the AA:DHA group (eFigure 3A, B, and C in Supplement 2).

## Discussion

Compared with the standard of care, enteral supplementation with a triglyceride oil containing AA and DHA during the first postnatal months resulted in a 50% reduction of severe ROP in infants with GA less than 28 weeks and increased overall mean serum levels of these fatty acids. Rates of BPD, PDA, and death were equal between the groups among infants with GA less than 27 weeks.

For infants with GA of 27 weeks, no increase in serum levels of AA and DHA were seen, and only 1 infant in the control group had severe ROP. However, BPD, PDA, and death rates were somewhat increased in the AA:DHA group. Mothers of infants born at 27 weeks’ GA in the control group were healthier than those in the AA:DHA group (any morbidity, 4 of 19 [21.1%] vs 14 of 23 [60.9%]). Low DHA levels after birth have been associated with chronic lung disease in preterm infants.^9^ Marc et al^26^ found a higher prevalence of BPD with maternal DHA supplementation among infants born at 27 to 28 weeks’ GA but not among those with a GA less than 27 weeks. This finding is in line with the results from our study. Considering the low risk of severe ROP among more mature preterm infants in a high-income neonatal setting, the potential harm of AA:DHA supplementation seems to outweigh the benefit for infants born at 27 weeks’ GA.

In our study, which included fewer but more immature infants, AA:DHA supplementation resulted in numerically comparable relative risks for BPD and death. However, regarding other neonatal morbidities, we found a numerically better outcome for severe IVH with AA:DHA, whereas Collins et al^19^ found the opposite with DHA only. We observed similar trends but somewhat attenuated effects for sepsis in our study.

With current nutrition regimes, preterm infants accumulate a deficit in both AA and DHA, and a previous study found an association between low neonatal AA levels and ROP.^9,17^ However, the provision of AA to infants in general is currently a matter of debate because regulatory standards on infant formula in the European Union stipulate that it needs to contain a higher fraction of DHA than in human milk, but no added AA.^27,28^ Studies on supplementation with LCPUFAs to improve preterm outcomes have mainly aimed to increase ω-3 fatty acids, especially DHA, and earlier studies reported improved visual and neurodevelopmental outcomes with such supplementation.^29,30^ The suggested mechanism of disease protection was the replacement of the more proinflammatory ω-6 fatty acids with the anti-inflammatory ω-3 fatty acids in membranes, thus decreasing inflammation.^12,31^

There is evidence that a balance between AA and DHA is important for early development. Both AA and DHA are selectively transferred from the mother to the fetus. When maternal DHA levels are low, the fetus receives higher circulating levels than the mother (biomagnification), but when maternal levels are high (maternal red blood cell DHA above approximately 6 g%), the fetal levels become lower than those of the mother (bioattenuation).^32^ For AA, biomagnification is independent of maternal AA status, and the fetal AA accretion rate is twice that of DHA.^21,32^ Animal studies indicate that AA and DHA compete for incorporation in tissues, including the brain.^33,34^ Increasing doses of DHA with a constant dose of AA in formula during the first year of life of infants born at term improved neurocognitive function up to the age of 6 years, but in the group with the highest DHA intake, AA in red blood cells decreased, and benefit was reduced.^35,36^ Feeding very preterm infants formulas with an AA to DHA ratio of 2:1 compared with 1:1 resulted in improved psychomotor development at 24 months of age.^37^

During gestation, the cord blood fatty acid profile is similar to that of the vascular endothelium, where AA is the main fatty acid, indicating a prominent role for AA in vascular development.^4^ After birth, the maternal AA and DHA source is interrupted, and the ω-6 fatty acid linoleic acid, which is involved in the formation of a barrier to reduce water loss through the skin, is provided in greater amounts than in utero, contributing to the postnatal reduction of fractions of AA and DHA.^38,39^ Both AA and DHA are present in equally high levels of approximately 10% of total fatty acids in bovine and porcine retinal vessels, while choroid and peripheral vessels contain more AA than DHA.^40,41^

The serum AA level was increased throughout the supplementation period; one might speculate that AA, with its abundance in blood vessels and growth-promoting effects, might have ameliorated normal retinal vascular growth. Infants who receive AA and DHA supplementation had higher serum DHA levels from 34 weeks’ PMA preceding the first diagnosis of severe ROP, which occurred at approximately 37 weeks’ PMA. Possible mechanisms for the preventive effect of DHA on severe ROP may be its anti-inflammatory effects and an inhibition of retinal endothelial cell proliferation and sprouting angiogenesis by the oxidized metabolite 4-hydroxy-docosahexaenoic acid observed in mice.^12^

The reduction in severe ROP among infants receiving AA and DHA supplementation was significant despite small but significant increases in serum levels. The distribution of enterally supplied AA and DHA in preterm infants is largely unknown, and the serum levels may not mirror the levels in organs. Rapid and selective incorporation in membranes may reduce serum fatty acid levels. Docosahexaenoic acid in triglycerides fed to 8-week-old rats was preferentially incorporated into adipose tissue and the heart but not in the brain, with a poor correlation between erythrocyte and brain ω-3 index.^42^

### Limitations and Strengths

This study has some limitations, including the lack of placebo, which meant that supplementary treatment was not masked to caregivers. However, screening ophthalmologists and those who analyzed fatty acids were unaware of whether the infant received AA and DHA supplementation or not. One also cannot exclude that ingredients in the oil other than AA and DHA may have affected the results. The strengths of the study include the multicenter design and the GA stratification with 3 GA groups, ensuring that immaturity is considered.

## Conclusions

Supplementing the diets of the most immature infants born at less than 27 weeks’ GA with an enteral lipid solution with AA to DHA ratio of 2:1 had no significant adverse effects and seems to be a promising intervention to prevent sight-threatening ROP and thereby reduce not only visual impartment and blindness but also exposure to potentially harmful therapies for severe ROP. Future studies need to focus on the optimal composition of fatty acids for preterm infants born at different stages of development. The goal should not be to replicate intrauterine fatty acid levels but to identify the fatty acid composition that results in the best health outcomes.

## Notes

Supplement 1. **Trial Protocol and Statistical Analysis Plan**

Supplement 2. **eAppendix 1.** Definitions of Study Outcomes and Exclusion Criteria **eAppendix 2.** Formulaid Content, AA:DHA Dose Adjusting Scheme and Diet **eAppendix 3.** Research Nurses Involved in the MDM Study **eAppendix 4.** Lipid Extraction and Fatty Acid Analysis **eTable 1.** Baseline Characteristics (PP Population) **eTable 2.** Nutrition (ITT Population) **eTable 3.** Sensitivity Analyses of Primary and Secondary Efficacy Variables (ITT Population) **eTable 4.** Analyses of Primary and Secondary Efficacy Variables (PP Population) **eTable 5.** Analyses of Primary Efficacy Variables Stratified by Gestational Age Randomization Strata (ITT Population) **eTable 6.** Analyses of the Secondary Efficacy Variables AA and DHA (ITT Population) **eTable 7.** Analyses of the Secondary Efficacy Variables AA and DHA (PP Population) **eTable 8.** Summary of Adverse Events (Safety Population) **eTable 9.** Adverse Events Including Pre-specified Terms and Studied Endpoints by Event and Category (Safety Population) **eTable 10.** Serious Adverse Events Including Pre-specified Terms and Studied Endpoints by Event and Category (Safety Population) **eTable 11.** Individual Causes of Death (n = 29) and Doses Taken **eFigure 1.** Serum Fractions of n-6 Arachidonic Acid (AA) and the n-3 Docosahexaenoic Acid (DHA) Stratified by Gestational Age Randomization Strata (ITT Population) **eFigure 2.** Cumulative Incidence Functions for Death in AA:DHA and Control Group (Safety Population) **eFigure 3.** Cumulative Incidence Functions for Death in AA:DHA and Control Group by Gestational Age (Safety Population) **eReferences.**

Supplement 3. **Data Sharing Statement**
