# Supplementary material for: Effect of Enteral Lipid Supplement on Severe Retinopathy of Prematurity: A Randomized Clinical Trial
Source: JAMA Pediatr. 2021 Feb 1;175(4):1–9. doi: 10.1001/jamapediatrics.2020.5653 (PMC7851754; doi:10.1001/jamapediatrics.2020.5653)
Supplement: Supplement 2. — eAppendix 1. Definitions of Study Outcomes and Exclusion Criteria eAppendix 2. Formulaid Content, AA:DHA Dose Adjusting Scheme and Diet eAppendix 3. Research Nurses Involved in the MDM Study eAppendix 4. Lipid Extraction and Fatty Acid Analysis eTable 1. Baseline Characteristics (PP Population) eTable 2. Nutrition (ITT Population) eTable 3. Sensitivity Analyses of Primary and Secondary Efficacy Variables (ITT Population) eTable 4. Analyses of Primary and Secondary Efficacy Variables (PP Population) eTable 5. Analyses of Primary Efficacy Variables Stratified by Gestational Age Randomization Strata (ITT Population) eTable 6. Analyses of the Secondary Efficacy Variables AA and DHA (ITT Population) eTable 7. Analyses of the Secondary Efficacy Variables AA and DHA (PP Population) eTable 8. Summary of Adverse Events (Safety Population) eTable 9. Adverse Events Including Pre-specified Terms and Studied Endpoints by Event and Category (Safety Population) eTable 10. Serious Adverse Events Including Pre-specified Terms and Studied Endpoints by Event and Category (Safety Population) eTable 11. Individual Causes of Death (n = 29) and Doses Taken eFigure 1. Serum Fractions of n-6 Arachidonic Acid (AA) and the n-3 Docosahexaenoic Acid (DHA) Stratified by Gestational Age Randomization Strata (ITT Population) eFigure 2. Cumulative Incidence Functions for Death in AA:DHA and Control Group (Safety Population) eFigure 3. Cumulative Incidence Functions for Death in AA:DHA and Control Group by Gestational Age (Safety Population) eReferences. [file jamapediatr-e205653-s002.pdf]

Supplementary Online Content

Hellström A, Nilsson AK, Wackernagel D, et al. Effect of enteral lipid supplement on severe retinopathy of prematurity: a randomized clinical trial. *JAMA Pediatr.* Published online February 1, 2021. doi:10.1001/jamapediatrics.2020.5653

eAppendix 1. Definitions of Study Outcomes and Exclusion Criteria
eAppendix 2. Formulaid Content, AA:DHA Dose Adjusting Scheme and Diet
eAppendix 3. Research Nurses Involved in the MDM Study
eAppendix 4. Lipid Extraction and Fatty Acid Analysis
eFigure 1. Serum Fractions of n-6 Arachidonic Acid (AA) and the n-3 Docosahexaenoic Acid (DHA) Stratified by Gestational Age Randomization Strata (ITT Population)
eFigure 2. Cumulative Incidence Functions for Death in AA:DHA and Control Group (Safety Population)
eFigure 3. Cumulative Incidence Functions for Death in AA:DHA and Control Group by Gestational Age (Safety Population)
eTable 1. Baseline Characteristics (PP Population)
eTable 2. Nutrition (ITT Population)
eTable 3. Sensitivity Analyses of Primary and Secondary Efficacy Variables (ITT Population)
eTable 4. Analyses of Primary and Secondary Efficacy Variables (PP Population)
eTable 5. Analyses of Primary Efficacy Variables Stratified by Gestational Age Randomization Strata (ITT Population)
eTable 6. Analyses of the Secondary Efficacy Variables AA and DHA (ITT Population)
eTable 7. Analyses of the Secondary Efficacy Variables AA and DHA (PP Population)
eTable 8. Summary of Adverse Events (Safety Population)
eTable 9. Adverse Events Including Pre-specified Terms and Studied Endpoints by Event and Category (Safety Population)
eTable 10. Serious Adverse Events Including Pre-specified Terms and Studied Endpoints by Event and Category (Safety Population)
eTable 11. Individual Causes of Death (n=29) and Doses Taken
eReferences.

This supplementary material has been provided by the authors to give readers additional information about their work.

eAppendix 1. Definitions of Study Outcomes and Exclusion Criteria

Bronchopulmonary Dysplasia

BPD was defined as the infant being treated with supplemental oxygen at 36 weeks postmenstrual age.

Intraventricular Hemorrhage

Cranial ultrasound was performed on postnatal days 3 and 7 and classified into either Grade I (germinal matrix hemorrhage), Grade II; (intraventricular hemorrhage without acute ventricular dilatation), Grade III (Intraventricular hemorrhage >50% of the ventricle with acute ventricular dilation) or Grade IV intraparenchymal lesion (periventricular hemorrhagic venous infarction).

Patent Ductus Arteriosus

Definition was based on need of treatment, surgically or medically (with paracetamol and/or ibuprofen) by clinical and functional echocardiography criteria.

Necrotizing Enterocolitis

The diagnosis of NEC was based on the criteria by Walsh et al in their modification of the original Bell´s criteria.[1] Stage 2A or greater was considered definite disease: Mild to moderate systemic signs, Additional intestinal signs (absent bowel sounds, abdominal tenderness), Specific radiologic signs (pneumatosis intestinalis or portal venous gas, Laboratory changes (metabolic acidosis, thrombocytopenia).

Walsh MC, Kliegmann RM. Necrotizing enterocolitis: treatment based on staging criteria. Pediatr Clin North Am 1986; 33:179-201

Growth

Weight, length and head circumference at birth and up to 40 weeks postmenstrual age was registered, standard deviation scores were calculated using the reference by Niklasson et al.[2]

Exclusion criteria

Detectable clinical gross malformation, known or suspected chromosomal abnormality, genetic disorder, or syndrome, clinically significant neuropathy, nephropathy, retinopathy, or other micro- or macrovascular disease requiring treatment or any other condition or therapy that might pose a risk to the subject or interfere with compliance or interfere with interpretation of results.

© 2021 Hellström A et al. *JAMA Pediatrics*.

eAppendix 2. Formulaid Content, AA:DHA Dose Adjusting Scheme and Diet

Formulaid™ 2:1 (ARA/DHA)		
Compound:	Oil	
Content:	Arachidonic acid (ARA)<br>Docosahexaenoic acid (DHA)<br>Ascorbyl palmitate<br>Tocopherols	240-290 mg/g (mean 265 mg/g)<br>120-150 mg/g (mean 135mg/g)<br>250 ppm<br>250-500 ppm
Density:	0.9 g/cm^3	

Fatty acid profile of Formulaid determined by GC-MS.

Fatty acid	Weight %
Myristic acid (14:0)	3.8
Palmitic acid (16:0)	10.0
Palmitoleic acid (16:1 n-7)	0.6
Margaric acid (17:0)	0.3
Stearic acid (18:0)	6.5
Oleic acid (18:1 n-9)	23.2
Linoleic acid (18:2 n-6)	4.8
γ-linolenic acid (18:3 n-6)	1.6
α-linolenic acid (18:3 n-3)	0.1
Arachidic acid (20:0)	0.6
Gondoic acid (20:1 n-9)	0.2

© 2021 Hellström A et al. *JAMA Pediatrics*.

Eicosadienoic acid (20:2 n-6)	0.4
Dihomo-γ-linolenic acid (20:3 n-6)	1.8
Arachidonic acid (20:4 n-6)	28.7
Eicosapentaenoic acid (20:5 n-3)	0.1
Behenic acid (22:0)	1.1
Docosapentaenoic acid (22:5 n-3)	0.2
Docosahexaenoic acid (22:6 n-3)	15.0
Lignoceric acid (24:0)	0.9

Supplementation with: DHA 50 mg/kg/day and ARA 100 mg/kg/day.

Administration starts at the third given meal and should be given at the same time each day. The trial emulsion was given after a small portion of human milk and before the main milk naso- or orogastric tube feeding. If feedings were stopped, the emulsion was withheld and restarted when feedings resumed. After gastric tube removal, AA:DHA supplementation was administered orally until 40 weeks PMA. The emulsion was quality tested and met quality thresholds throughout.

Dose increase by 0.1 ml according to below dosing scheme. Maximum dose 1 ml/day. The dose is calculated on birthweight until the weight has increased above the birthweight, thereafter it is calculated on actual weight.

Enteral intake	Breastmilk and other formulas	Only 100% PreNAN Discharge<br>(contains 14.4 mg/100ml ARA and 14.4 mg/100 ml DHA)
Formulaid™ dos	0,39 ml/kg/day once daily	0.33 ml/kg/day once daily

	Weights (gram) for dose increase	
Formulaid (ml)	Nutrition: breastmilk, other formulas or partly PreNAN	Nutrition: 100% PreNAN Discharge

© 2021 Hellström A et al. *JAMA Pediatrics*.

	Discharge	
0.1	260 g	-
0.2	520 g	-
0.3	780 g	-
0.4	1040 g	1215 g
0.5	1300 g	1520 g
0.6	1560 g	1825 g
0.7	1820 g	2130 g
0.8	2080 g	2435 g
0.9	2340 g	2740 g
1.0	2600 g	g

Diet

Clinoleic was used as parenteral lipid emulsion.

Formulas and fortifiers used, as a compliment to breastmilk if needed, were PreNAN Discharge, FM 85 or Nutriprem according to local guidelines.

Calogen or Liquigen were used as energy enrichments.

© 2021 Hellström A et al. *JAMA Pediatrics*.

eAppendix 3. Research Nurses Involved in the MDM Study

Carola Pfeiffer Mosesson[1], Ann-Charlotte Andersson[1], Cathrine Ragoonan[1], Jonathan Servello[1], Margareta Gebka[2], Ann-Cathrine Berg[2], Linda Nilsson[2], Camilla Halzius[3], Michaela Melakari[3], Lena Legnevall[3], Therése Kjellin[3]

[1]Section for Ophthalmology, Department of Clinical Neuroscience, Institute of Neuroscience and Physiology, Sahlgrenska Academy, University of Gothenburg, Gothenburg, Sweden[1]

[2]Department of Pediatrics of Clinical Sciences Lund, Lund University and Skane University Hospital, Lund Sweden[2]

[3]Department of Neonatology, Karolinska Institute and University Hospital, Stockholm, Sweden[3]

© 2021 Hellström A et al. *JAMA Pediatrics*.

eAppendix 4. Lipid Extraction and Fatty Acid Analysis

Blood samples were taken at birth (cord blood) at postnatal days 1, 3, 7, 14, and postnatal week 4 and 6 and then at PMA 30, 32, 34, 36, and 40 weeks. Extraction of lipids from serum and analysis of phospholipid fatty acids were as previously described but with minor modifications (3). Briefly, in a glass tube, 25 µl serum was spiked with 1,2-dinonadecanoyl-sn-glycero-3-phosphatidylcholine (19:0 PC) corresponding to 2 µg methyl nonadecanoate. Samples were lyophilized and extracted three times in chloroform:methanol (2:1) containing 0.05 (w/v)% butylated hydroxytoluene. The total lipid extract was evaporated to dryness under N_2 gas, reconstituted in chloroform, and then fractionated on a single Sep-Pak 1 cc aminopropyl cartridge (Waters Corporation, Milford, MA, US). The cartridge was activated with hexane before the sample was loaded, then washed with chloroform:isopropanol (2:1) follow by 2% acetic acid in diethyl ether, and phospholipids were eluted with methanol. Fatty acids methyl esters (FAMEs) were prepared using 3M HCl in methanol (Sigma-Aldrich, Merck KGaA, Darmstadt, Germany) as a derivatizing agent at 80°C over four hours. After cooling, hexane was added and the upper organic layer containing FAMEs was collected, washed with water, dried under N_2, and finally dissolved in 75 µl hexane.

One microliter of the sample was injected in pulsed (40 psi for 1 min) splitless mode on an Agilent 7820 gas chromatograph coupled to an Agilent 5975 mass spectrometer (Agilent Technologies, Palo Alto, CA, US) using helium as the carrier gas at a constant flow of 0.62 mL min^{-1}. Separation of FAMEs was achieved on a 0.25 mm DB-23 capillary column (Agilent Technologies) using the previously described oven conditions (4). Identification and quantification of FAMEs were performed by comparison to serial diluted authentic standards (4).

Sample preparation and GC FAME analyses were performed continuously through the trial as infants completed the study period. A control sample was included in each lipid extraction batch (approximately 15-20 study samples per batch) to monitor for variability in the method. The coefficient of variance for the control sample over the whole study period was 7.6% for AA (mean 7.2 mol%) and 12.8% for DHA (mean 3.7 mol%)(n=107).

© 2021 Hellström A et al. *JAMA Pediatrics*.

eFigure 1. Serum Fractions of n-6 Arachidonic Acid (AA) and the n-3 Docosahexaenoic Acid (DHA) Stratified by Gestational Age Randomization Strata (ITT Population). GA at birth <25 weeks, AA:DHA n=37 and controls n=39; GA at birth 25-26 weeks, AA:DHA n=41 and controls n=47; GA at birth 27 weeks, AA:DHA n= 23 and controls n=19.

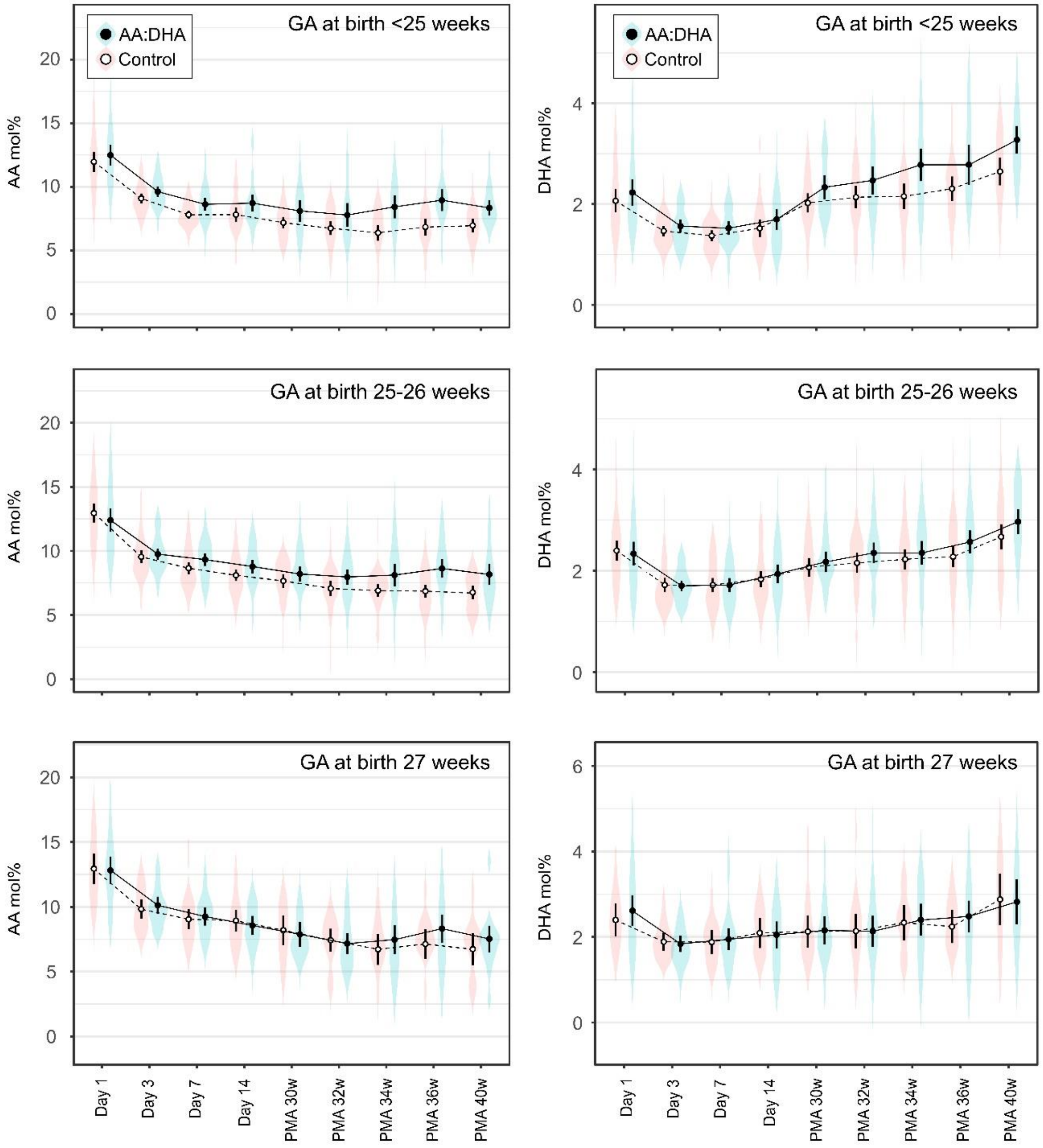

© 2021 Hellström A et al. *JAMA Pediatrics*.

eFigure 2. Cumulative Incidence Functions for Death in AA:DHA and Control Group (Safety Population)

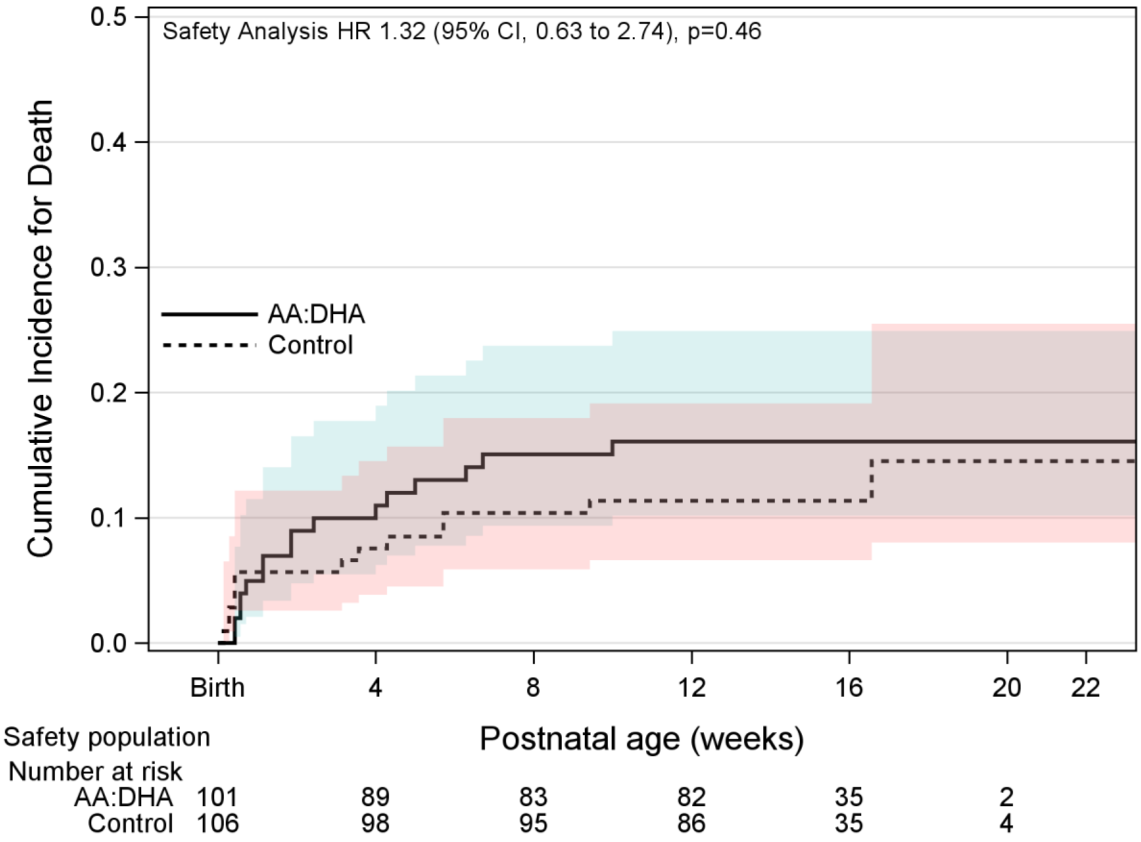

© 2021 Hellström A et al. *JAMA Pediatrics*.

eFigure 3. Cumulative Incidence Functions for Death in AA:DHA and Control Group by Gestational Age (Safety Population)
a) GA <25 Weeks b) GA 25-26 Weeks c) GA 27 Weeks.

a)

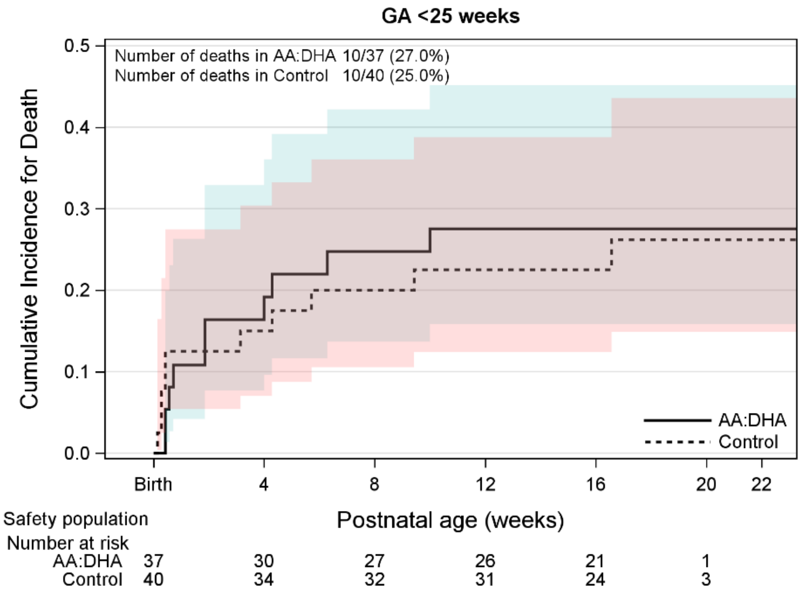

b)

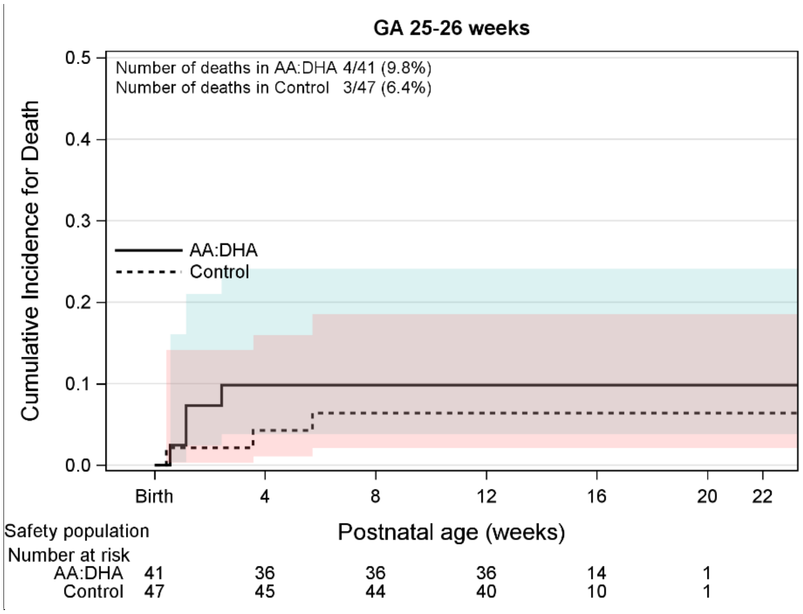

© 2021 Hellström A et al. *JAMA Pediatrics*.

c)

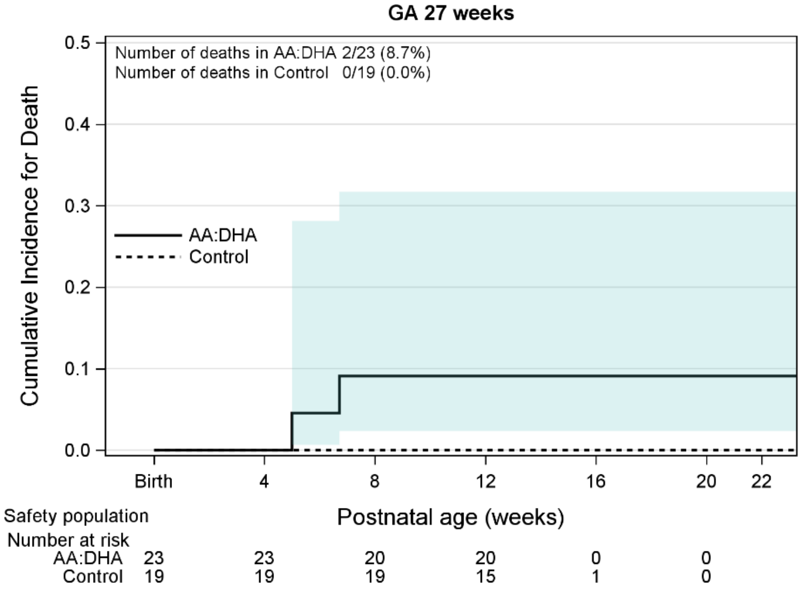

© 2021 Hellström A et al. *JAMA Pediatrics*.

eTable 1. Baseline Characteristics (PP Population)

	Actual Group: Control (n=94)	Actual Group: AA:DHA (n=81)
**Gestational age (weeks)**	25.6 (1.4)<br>25.8 (22.9; 27.9)<br>n=94	25.6 (1.5)<br>25.6 (22.6; 27.9)<br>n=81
**Gestational age at birth**		
**<25 weeks**	31 (33.0%)	25 (30.9%)
**25-26 weeks**	44 (46.8%)	37 (45.7%)
**≥27 weeks**	19 (20.2%)	19 (23.5%)
**Female sex**	43 (45.7%)	33 (40.7%)
**Birth weight (g)**	795 (195)<br>775 (425; 1330)<br>n=94	817 (205)<br>790 (455; 1345)<br>n=81
**Birth weight (SDS)**	-0.771 (1.156)<br>-0.498 (-4.058; 1.222)<br>n=94	-0.670 (1.249)<br>-0.368 (-5.058; 1.744)<br>n=81
**Birth length (cm)**	33.0 (2.8)<br>33.0 (28.5; 41.0)<br>n=77	32.9 (2.6)<br>33.0 (27.0; 38.0)<br>n=71
**Birth length (SDS)**	-1.27 (1.54)<br>-1.03 (-4.70; 2.89)<br>n=77	-1.29 (1.60)<br>-1.08 (-6.76; 1.42)<br>n=71
**Birth head circumference (cm)**	23.3 (1.8)<br>23.3 (20.2; 27.5)<br>n=92	23.3 (1.9)<br>23.3 (18.7; 27.5)<br>n=80
**Birth head circumference (SDS)**	12.6 (2.4)<br>12.4 (7.4; 17.5)<br>n=92	12.8 (2.6)<br>12.6 (8.0; 19.9)<br>n=80
**Baseline AA (mol%) in serum**	2.26 (0.65)<br>2.27 (0.77; 4.39)<br>n=92	2.36 (0.80)<br>2.18 (1.09; 4.78)<br>n=80
**Baseline DHA (mol%) in serum**	2.22 (0.66)<br>2.23 (0.77; 4.39)<br>n=92	2.32 (0.83)<br>2.09 (1.09; 4.78)<br>n=80
**Plurality**		
**Single**	75 (79.8%)	66 (81.5%)
**Twin**	18 (19.1%)	12 (14.8%)
**Triplet**	1 (1.1%)	3 (3.7%)
**Center**		
**Gothenburg**	33 (35.1%)	32 (39.5%)
**Stockholm**	28 (29.8%)	23 (28.4%)

© 2021 Hellström A et al. *JAMA Pediatrics*.

	Actual Group: Control (n=94)	Actual Group: AA:DHA (n=81)
**Lund**	33 (35.1%)	26 (32.1%)
**Mother's age (years)**	31.9 (5.0)<br>31.1 (22.4; 46.2)<br>n=93	32.3 (5.3)<br>33.0 (16.2; 41.2)<br>n=79
**Parity (number of child in order)**	1.65 (0.90)<br>1.00 (1.00; 5.00)<br>n=93	1.67 (0.88)<br>1.00 (1.00; 5.00)<br>n=78
**Caesarean section**	56 (59.6%)	52 (64.2%)
**Mothers with any medical history**	30 (31.9%)	41 (50.6%)
**Mothers with diabetes**	0 (0.0%)	1 (1.2%)
**Mothers with hypertension**	13 (13.8%)	6 (7.4%)

For categorical variables n (%) is presented.
For continuous variables Mean (SD) / Median (Min; Max) / n= is presented.
SDS = standard deviation score using Niklasson and Albertsson-Wikland reference from 2008 [2]; AA = arachidonic acid; DHA = docosahexaeonic acid

© 2021 Hellström A et al. *JAMA Pediatrics*.

eTable 2. Nutrition (ITT Population)

	Randomized Group: Control (n=105)				Randomized Group: AA:DHA (n=101)			
**Total parenteral and enteral intake**	**Week 1**<br>**n=100**	**Week 2**<br>**n=100**	**Week 3**<br>**n=100**	**Week 4**<br>**n=100**	**Week 1**<br>**n=95**	**Week 2**<br>**n=90**	**Week 3**<br>**n=88**	**Week 4**<br>**n=87**
**Fluid (ml/kg/d)**	137 (16)<br>134<br>(93;186)	167 (11)<br>166<br>(146;205)	168 (13)<br>168<br>(124;199)	167 (14)<br>166<br>(90;202)	138 (19)<br>138<br>(83;192)	171 (14)<br>169<br>(144;220)	171 (14)<br>172<br>(132;210)	170 (14)<br>169<br>(113;213)
**Energy (kcal/kg/d)**	86 (9)<br>86<br>(65;111)	119 (15)<br>119<br>(78;150)	130 (21)<br>133<br>(60;175)	135 (21)<br>139<br>(67;198)	87 (12)<br>88<br>(37;120)	123 (19)<br>122<br>(85;186)	132 (21)<br>133<br>(85;187)	137 (22)<br>139<br>(76;210)
**Protein (g/kg/d)**	2.9 (0.4)<br>2.9<br>(2.0;3.9)	3.8 (0.6)<br>3.8<br>(2.1;5.8)	4.0 (0.6)<br>4.0<br>(2.1;5.9)	4.0 (0.6)<br>4.0<br>(2.0;5.1)	2.9 (0.5)<br>2.9<br>(1.2;4.1)	3.8 (0.6)<br>3.8<br>(2.5;5.8)	3.9 (0.6)<br>3.9<br>(2.7;5.8)	3.9 (0.6)<br>3.9<br>(2.0;5.9)
**Carbohydrates (g/kg/d)**	10.3 (1.2)<br>10.4<br>(7.5;13.3)	12.8 (1.3)<br>12.7<br>(8.7;16.6)	13.5 (1.5)<br>13.6<br>(9.5;17.3)	13.9 (1.7)<br>14.1<br>(8.7;19.3)	10.3 (1.5)<br>10.1<br>(4.8;14.1)	13.3 (1.9)<br>13.0<br>(9.7;21.7)	13.9 (2.1)<br>13.6<br>(9.1;22.9)	13.9 (2.0)<br>13.9<br>(6.9;21.0)
**Lipids (g/kg/d)**	3.6 (0.8)<br>3.5<br>(1.3;5.5)	5.6 (1.4)<br>5.6<br>(2.4;8.6)	6.4 (1.9)<br>6.6<br>(1.4;10.5)	6.7 (1.9)<br>6.9<br>(2.4;12.4)	3.7 (0.8)<br>3.8<br>(1.1;5.9)	5.8 (1.6)<br>5.6<br>(1.8;12.4)	6.4 (1.9)<br>6.6<br>(1.7;12.7)	6.9 (2.0)<br>6.9<br>(1.5;12.6)
For continuous variables Mean (SD) / Median (Min; Max) is presented.								

© 2021 Hellström A et al. *JAMA Pediatrics*.

eTable 3. Sensitivity Analyses of Primary and Secondary Efficacy Variables (ITT Population)

	Measure	Randomized Group: Control (n=105)	Randomized Group: AA:DHA (n=101)	Comparison: AA:DHA vs Control
Primary Outcome				
**Severe ROP**	CIF (95% CI)<br>at 20 weeks	0.38 (0.26 - 0.49)	0.19 (0.11 - 0.28)	HRs (95% CI)<br>0.44 (0.24 - 0.82)<br>p=0.006
Secondary Outcomes				
**BPD**	n/N (%)<br>event rate (95% CI)[a]	59/105 (56.2%)<br>57.2 (44.3 - 73.8)	65/101 (64.4%)<br>69.8 (54.8 - 89.1)	RR (95% CI)<br>1.22 (0.86 - 1.74)<br>p=0.27
**IVH**				
**No IVH**	n (%)	62 (59.0%)	58 (57.4%)	
**Grade I**	n (%)	11 (10.5%)	16 (15.8%)	
**Grade II**	n (%)	14 (13.3%)	17 (16.8%)	
**Grade III**	n (%)	5 (4.8%)	6 (5.9%)	
**Grade IV**	n (%)	13 (12.4%)	4 (4.0%)	
**PDA**	n (%)	57 (54.3%)	66 (65.3%)	
**NEC**	CIF (95% CI)<br>at 20 weeks	0.11 (0.06 - 0.17)	0.10 (0.05 - 0.17)	HRs (95% CI)<br>0.94 (0.40 - 2.20)

[a] per 1000 person weeks

ROP = retinopathy of prematurity; BPD = bronchopulmonary dysplasia; IVH = intraventricular haemorrhage; PDA = patent ductus arteriosus; NEC = necrotizing enterocolitis; CIF = cumulative incidence function; CI = confidence intervals; HRs = sub-distribution hazard ratio obtained from the survival analysis according to Fine and Gray adjusting for death as competing risk; RR = relative risk;

© 2021 Hellström A et al. *JAMA Pediatrics*.

eTable 4. Analyses of Primary and Secondary Efficacy Variables (PP Population)

	Measure	Actual Group: Control (n=94)	Actual Group: AA:DHA (n=81)	Comparison: AA:DHA vs Control
Primary Outcome				
**Severe ROP**	n/N (%)<br>event rate (95% CI)[a]	35/94 (37.2%)<br>13.7 (6.7 - 27.9)	15/81 (18.5%)<br>6.7 (3.0 - 14.9)	RR (95% CI)<br>0.49 (0.27 - 0.89)<br>p=0.020
**ROP stage[b]**				
**No ROP**	n (%)	37 (39.4%)	33 (40.7%)	
**ROP Stage 1-2**	n (%)	22 (23.4%)	33 (40.7%)	
**ROP Stage 3 or Type 1**	n (%)	35 (37.2%)	15 (18.5%)	
Secondary Outcomes				
**AA (mol%) overall[c]**	LS means (95% CI)	7.73 (7.49 - 7.97)	8.54 (8.26 - 8.83)	Difference in LS means (95% CI)<br>-0.81 (-1.18 - -0.44)<br>p<0.001
**DHA (mol%) overall[c]**	LS means (95% CI)	2.08 (2.00 - 2.16)	2.24 (2.14 - 2.33)	Difference in LS means (95% CI)<br>-0.16 (-0.28 - -0.04)<br>p=0.012
**BPD**	n/N (%)<br>event rate (95% CI)[a]	48/94 (51.1%)<br>48.1 (36.2 - 63.8)	45/81 (55.6%)<br>52.8 (39.4 - 70.8)	RR (95% CI)<br>1.10 (0.73 - 1.65)<br>p=0.65
**IVH**				
**No IVH**	n (%)	58 (61.7%)	50 (61.7%)	
**Grade I**	n (%)	10 (10.6%)	13 (16.0%)	
**Grade II**	n (%)	12 (12.8%)	11 (13.6%)	
**Grade III**	n (%)	5 (5.3%)	4 (4.9%)	
**Grade IV**	n (%)	9 (9.6%)	3 (3.7%)	
**PDA**	n (%)	46 (48.9%)	49 (60.5%)	
**NEC**	n/N (%)<br>event rate (95% CI)[a]	10/94 (10.6%)<br>7.5 (4.0 - 14.0)	4/81 (4.9%)<br>3.3 (1.2 - 8.7)	RR (95% CI)<br>0.44 (0.14 - 1.39)
**Weight (SDS) overall[c]**	LS means (95% CI)	-1.63 (-1.77 - -1.49)	-1.75 (-1.92 - -1.58)	Difference in LS means (95% CI)<br>0.12 (-0.10 - 0.34)
**Length (SDS) overall[c]**	LS means (95% CI)	-2.91 (-3.15 - -2.66)	-2.94 (-3.21 - -2.67)	Difference in LS means (95% CI)<br>0.03 (-0.32 - 0.39)
**Head Circumference (SDS) overall[c]**	LS means (95% CI)	-1.46 (-1.62 - -1.31)	-1.47 (-1.65 - -1.30)	Difference in LS means (95% CI)<br>0.01 (-0.23 - 0.25)

© 2021 Hellström A et al. *JAMA Pediatrics*.

	Measure	Actual Group: Control (n=94)	Actual Group: AA:DHA (n=81)	Comparison: AA:DHA vs Control

[a] per 1000 person weeks

[b] descriptively only, not part of the planned analyses

[c] overall effect was analyzed using mixed models for repeated measures including treatment group, visit, interaction between treatment group and visit, and at birth value as fixed effects. Unstructured covariance pattern was applied by treatment group.

ROP = retinopathy of prematurity; AA = arachidonic acid; DHA = docosahexaenoic acid; BPD = bronchopulmonary dysplasia; IVH = intraventricular haemorrhage; PDA = patent ductus arteriosus; NEC = necrotizing enterocolitis; SDS = standard deviation score using Niklasson and Albertsson-Wikland reference from 2008 [2]; CI = confidence intervals; RR = relative risk; LS = least square;

© 2021 Hellström A et al. *JAMA Pediatrics*.

eTable 5. Analyses of Primary Efficacy Variables Stratified by Gestational Age Randomization Strata (ITT Population)

	GA randomization strata	Measure	Randomized Group: Control (n=105)	Randomized Group: AA:DHA (n=101)	Comparison: AA:DHA vs Control
Primary Outcome					
**Severe ROP**	**<25 weeks**	n/N (%)<br>event rate (95% CI)[a]	22/39 (56.4%)<br>49.0 (32.3 - 74.4)	9/37 (24.3%)<br>20.7 (10.8 - 39.8)	RR (95% CI)<br>0.42 (0.19 - 0.92)
**Severe ROP**	**25-26 weeks**	n/N (%)<br>event rate (95% CI)[a]	12/47 (25.5%)<br>18.7 (10.6 - 33.0)	7/41 (17.1%)<br>13.0 (6.2 - 27.3)	RR (95% CI)<br>0.69 (0.27 - 1.76)
**Severe ROP**	**≥27 weeks**	n/N (%)<br>event rate (95% CI)[a]	1/19 (5.3%)<br>4.1 (0.6 - 29.3)	0/23 (0.0%)	--
Secondary Outcomes					
**AA**	**<25 weeks**	LS means (95% CI)	7.47 (7.13 - 7.80)	8.52 (7.99 - 9.05)	Difference in LS means (95% CI)<br>1.05 (0.44 - 1.66)
**AA**	**25-26 weeks**	LS means (95% CI)	7.69 (7.30 - 8.07)	8.70 (8.30 - 9.09)	Difference in LS means (95% CI)<br>1.01 (0.46 - 1.56)
**AA**	**≥27 weeks**	LS means (95% CI)	7.90 (7.21 - 8.58)	8.24 (7.62 - 8.86)	Difference in LS means (95% CI)<br>0.34 (-0.58 - 1.26)
**DHA**	**<25 weeks**	LS means (95% CI)	1.98 (1.87 - 2.10)	2.22 (2.06 - 2.38)	Difference in LS means (95% CI)<br>0.24 (0.05 - 0.43)
**DHA**	**25-26 weeks**	LS means (95% CI)	2.07 (1.94 - 2.21)	2.22 (2.10 - 2.34)	Difference in LS means (95% CI)<br>0.15 (-0.03 - 0.32)
**DHA**	**≥27 weeks**	LS means (95% CI)	2.24 (2.04 - 2.43)	2.15 (1.97 - 2.33)	Difference in LS means (95% CI)<br>-0.09 (-0.35 - 0.18)
**BPD**	**<25 weeks**	n/N (%)<br>event rate (95% CI)[a]	23/39 (59.0%)<br>57.0 (37.9 - 85.8)	21/37 (56.8%)<br>59.4 (38.7 - 91.1)	RR (95% CI)<br>1.04 (0.58 - 1.88)
**BPD**	**25-26 weeks**	n/N (%)<br>event rate (95% CI)[a]	21/47 (44.7%)<br>45.5 (29.7 - 69.8)	17/41 (41.5%)<br>44.5 (27.7 - 71.6)	RR (95% CI)<br>0.98 (0.52 - 1.86)
**BPD**	**≥27 weeks**	n/N (%)<br>event rate (95% CI)[a]	4/19 (21.1%)<br>23.9 (9.0 - 63.8)	10/23 (43.5%)<br>51.2 (27.6 - 95.2)	RR (95% CI)<br>2.14 (0.67 - 6.82)
**IVH**	**<25 weeks**				
**Grade 0**		n (%)	16 (41.0%)	17 (45.9%)	
**Grade 1**		n (%)	6 (15.4%)	4 (10.8%)	
**Grade 2**		n (%)	7 (17.9%)	10 (27.0%)	
**Grade 3**		n (%)	3 (7.7%)	3 (8.1%)	
**Grade 4**		n (%)	7 (17.9%)	3 (8.1%)	
**IVH**	**25-26 weeks**				

© 2021 Hellström A et al. *JAMA Pediatrics*.

**Grade 0**		n (%)	30 (63.8%)	23 (56.1%)	
**Grade 1**		n (%)	4 (8.5%)	10 (24.4%)	
**Grade 2**		n (%)	6 (12.8%)	4 (9.8%)	
**Grade 3**		n (%)	2 (4.3%)	3 (7.3%)	
**Grade 4**		n (%)	5 (10.6%)	1 (2.4%)	
**IVH**	**≥27 weeks**				
**Grade 0**		n (%)	17 (89.5%)	18 (78.3%)	
**Grade 1**		n (%)	1 (5.3%)	2 (8.7%)	
**Grade 2**		n (%)	1 (5.3%)	3 (13.0%)	
**Grade 3**		n (%)	0 (0.0%)	0 (0.0%)	
**Grade 4**		n (%)	0 (0.0%)	0 (0.0%)	
**PDA**	**<25 weeks**	n (%)	29 (74.4%)	25 (67.6%)	
**PDA**	**25-26 weeks**	n (%)	20 (42.6%)	19 (46.3%)	
**PDA**	**≥27 weeks**	n (%)	2 (10.5%)	9 (39.1%)	
**NEC**	**<25 weeks**	n/N (%)	3/39 (7.7%)	5/37 (13.5%)	RR (95% CI)
		event rate (95% CI)a	5.7 (1.8 - 17.6)	11.1 (4.6 - 26.7)	1.96 (0.47 - 8.20)
**NEC**	**25-26 weeks**	n/N (%)	5/47 (10.6%)	3/41 (7.3%)	RR (95% CI)
		event rate (95% CI)[a]	8.1 (3.4 - 19.4)	5.3 (1.7 - 16.5)	0.66 (0.16 - 2.75)
**NEC**	**≥27 weeks**	n/N (%)	3/19 (15.8%)	2/23 (8.7%)	RR (95% CI)
		event rate (95% CI)[a]	14.2 (4.6 - 44.0)	7.3 (1.8 - 29.1)	0.51 (0.09 - 3.07)

[a] per 1000 person weeks

GA=gestational age; ROP = retinopathy of prematurity; AA = arachidonic acid; DHA = docosahexaenoic acid; BPD = bronchopulmonary dysplasia; IVH = intraventricular haemorrhage; PDA = patent ductus arteriosus; NEC = necrotizing enterocolitis; SDS = standard deviation score; CI = confidence intervals; RR = relative risk; LS = least square;

© 2021 Hellström A et al. *JAMA Pediatrics*.

eTable 6. Analyses of the Secondary Efficacy Variables AA and DHA (ITT Population)

	AA (mol%)					DHA (mol%)				
	Raw data		MMRM			Raw data		MMRM		
Time point	Randomized group: Control Mean (SD) Median (Min-Max) n	Randomized group: AA:DHA Mean (SD) Median (Min-Max) n	Randomized group: Control LS means (95% CI)	Randomized group: AA:DHA LS means (95% CI)	Difference (AA:DHA - Control) in LS means (95% CI) p-value	Randomized group: Control Mean (SD) Median (Min-Max) n	Randomized group: AA:DHA Mean (SD) Median (Min-Max) n	Randomized group: Control LS means (95% CI)	Randomized group: AA:DHA LS means (95% CI)	Difference (AA:DHA - Control) in LS means (95% CI) p-value
**Overall (analysis)**			8.18 (7.94 - 8.43)	8.77 (8.50 - 9.04)	0.59 (0.24 - 0.93) p<0.001			2.12 (2.03 - 2.21)	2.17 (2.07 - 2.27)	0.05 (-0.06 - 0.17) p=0.36
**Cord blood**	15.25 (2.93) 15.22 (9.08 - 21.08) n=32	15.57 (2.90) 16.02 (6.79 - 20.07) n=33				2.43 (0.69) 2.43 (1.21 - 3.57) n=32	2.85 (0.97) 2.82 (0.64 - 5.38) n=33			
**Day 0**	12.57 (2.44) 12.33 (7.45 - 17.53) n=101	12.59 (2.64) 12.44 (8.01 - 19.87) n=99	12.59 (12.11 - 13.07)	12.57 (12.04 - 13.09)	-0.03 (-0.73 - 0.68)	2.28 (0.68) 2.28 (0.77 - 4.39) n=101	2.37 (0.82) 2.17 (1.09 - 4.78) n=99	2.28 (2.15 - 2.41)	2.37 (2.21 - 2.54)	0.09 (-0.11 - 0.30)
**Day 3**	9.40 (1.48) 9.05 (6.07 - 14.21) n=95	9.75 (1.33) 9.59 (6.94 - 13.10) n=94	9.43 (9.14 - 9.73)	9.79 (9.52 - 10.06)	0.35 (-0.05 - 0.76)	1.65 (0.42) 1.57 (0.88 - 3.18) n=95	1.66 (0.37) 1.63 (0.96 - 2.60) n=94	1.66 (1.58 - 1.75)	1.69 (1.61 - 1.77)	0.02 (-0.09 - 0.14)
**Day 7**	8.46 (1.49) 8.13 (5.89 - 14.17) n=94	9.05 (1.49) 9.03 (5.22 - 12.58) n=86	8.43 (8.12 - 8.73)	9.03 (8.73 - 9.34)	0.61 (0.18 - 1.03)	1.64 (0.47) 1.57 (0.79 - 2.95) n=94	1.71 (0.52) 1.63 (0.67 - 3.87) n=86	1.62 (1.53 - 1.71)	1.71 (1.60 - 1.81)	0.08 (-0.05 - 0.22)
**Day 14**	8.11 (1.59) 7.79 (4.58 - 12.71) n=91	8.67 (1.66) 8.47 (4.87 - 13.71) n=84	8.12 (7.80 - 8.45)	8.68 (8.32 - 9.03)	0.55 (0.07 - 1.03)	1.76 (0.55) 1.69 (0.76 - 3.17) n=91	1.87 (0.62) 1.82 (0.76 - 3.45) n=84	1.77 (1.66 - 1.88)	1.89 (1.75 - 2.03)	0.13 (-0.05 - 0.30)
**Week 4**	7.98 (1.68) 7.83 (4.22 - 12.45) n=44	8.22 (1.73) 8.09 (3.97 - 13.29) n=35				1.85 (0.59) 1.76 (0.81 - 3.90) n=44	2.11 (0.52) 2.05 (1.23 - 3.09) n=35			
**Week 6**	7.92 (0.91) 8.42 (6.79 - 8.68) n=5	7.97 (1.59) 7.30 (6.12 - 10.77) n=9				1.96 (0.21) 1.93 (1.82 - 2.32) n=5	2.14 (0.65) 1.99 (1.36 - 3.26) n=9			

© 2021 Hellström A et al. *JAMA Pediatrics*.

	AA (mol%)					DHA (mol%)				
	Raw data		MMRM			Raw data		MMRM		
Time point	Randomized group: Control Mean (SD) Median (Min-Max) n	Randomized group: AA:DHA Mean (SD) Median (Min-Max) n	Randomized group: Control LS means (95% CI)	Randomized group: AA:DHA LS means (95% CI)	Difference (AA:DHA - Control) in LS means (95% CI) p-value	Randomized group: Control Mean (SD) Median (Min-Max) n	Randomized group: AA:DHA Mean (SD) Median (Min-Max) n	Randomized group: Control LS means (95% CI)	Randomized group: AA:DHA LS means (95% CI)	Difference (AA:DHA - Control) in LS means (95% CI) p-value
**PMA 30 weeks**	7.59 (1.51)<br>7.59 (3.27 - 10.66)<br>n=77	8.14 (1.69)<br>8.03 (5.00 - 12.52)<br>n=71	7.61 (7.26 - 7.96)	8.08 (7.70 - 8.47)	0.47 (-0.04 - 0.98)	2.11 (0.58)<br>2.01 (0.92 - 4.14)<br>n=77	2.23 (0.60)<br>2.09 (0.76 - 3.89)<br>n=71	2.07 (1.94 - 2.20)	2.21 (2.08 - 2.35)	0.14 (-0.04 - 0.33)
**PMA 32 weeks**	7.11 (1.64)<br>7.15 (1.63 - 10.74)<br>n=83	7.69 (2.00)<br>7.62 (2.93 - 12.64)<br>n=76	7.04 (6.69 - 7.39)	7.68 (7.24 - 8.12)	0.63 (0.08 - 1.19)	2.20 (0.66)<br>2.27 (0.68 - 4.47)<br>n=83	2.34 (0.70)<br>2.25 (0.67 - 4.28)<br>n=76	2.14 (2.01 - 2.28)	2.32 (2.17 - 2.47)	0.17 (-0.03 - 0.37)
**PMA 34 weeks**	6.97 (1.62)<br>7.08 (2.29 - 11.60)<br>n=77	8.14 (2.34)<br>8.22 (3.23 - 13.70)<br>n=72	6.71 (6.36 - 7.07)	8.08 (7.56 - 8.61)	1.37 (0.74 - 2.00)	2.24 (0.64)<br>2.29 (0.93 - 3.69)<br>n=77	2.49 (0.79)<br>2.54 (0.72 - 4.30)<br>n=72	2.20 (2.07 - 2.34)	2.49 (2.32 - 2.67)	0.29 (0.07 - 0.51)
**PMA 36 weeks**	7.08 (1.71)<br>6.98 (2.61 - 11.60)<br>n=77	8.72 (2.22)<br>8.72 (3.30 - 13.91)<br>n=66	6.89 (6.53 - 7.25)	8.59 (8.10 - 9.07)	1.70 (1.10 - 2.30)	2.32 (0.62)<br>2.35 (0.80 - 3.99)<br>n=77	2.66 (0.81)<br>2.64 (0.81 - 4.42)<br>n=66	2.28 (2.15 - 2.42)	2.61 (2.42 - 2.79)	0.33 (0.10 - 0.55)
**PMA 40 weeks**	6.84 (1.50)<br>6.99 (3.60 - 11.05)<br>n=65	8.02 (1.94)<br>7.75 (2.94 - 13.52)<br>n=68	6.83 (6.47 - 7.18)	7.98 (7.52 - 8.45)	1.16 (0.58 - 1.74)	2.75 (0.78)<br>2.79 (1.36 - 4.60)<br>n=65	3.01 (0.77)<br>2.97 (1.01 - 4.69)<br>n=68	2.70 (2.52 - 2.88)	3.01 (2.83 - 3.18)	0.31 (0.06 - 0.56)

Analyses are performed using Mixed Models for Repeated Measures (MMRM) data. Overall analyses were including treatment group and time points as fixed effects, and by time point analyses additionally including also an interaction term between treatment group and time points.

© 2021 Hellström A et al. *JAMA Pediatrics*.

eTable 7. Analyses of the Secondary Efficacy Variables AA and DHA (PP Population)

	AA (mol%)					DHA (mol%)				
	Raw data		MMRM			Raw data		MMRM		
Time point	Actual group: Control Mean (SD) Median (Min-Max) n	Actual group: AA:DHA Mean (SD) Median (Min-Max) n	Actual group: Control LS means (95% CI)	Actual group: AA:DHA LS means (95% CI)	Difference (AA:DHA - Control) in LS means (95% CI) p-value	Actual group: Control Mean (SD) Median (Min-Max) n	Actual group: AA:DHA Mean (SD) Median (Min-Max) n	Actual group: Control LS means (95% CI)	Actual group: AA:DHA LS means (95% CI)	Difference (AA:DHA - Control) in LS means (95% CI) p-value
**Overall (analysis)**			8.26 (8.01 - 8.51)	8.88 (8.60 - 9.17)	-0.62 (-0.97 - -0.27) p<0.001			2.13 (2.03 - 2.22)	2.19 (2.08 - 2.29)	-0.06 (-0.18 - 0.06) p=0.31
**Cord blood**	15.25 (2.93) 15.22 (9.08 - 21.08) n=32	15.70 (2.36) 16.05 (10.93 - 20.07) n=26				2.43 (0.69) 2.43 (1.21 - 3.57) n=32	2.83 (0.91) 2.71 (1.47 - 5.38) n=26			
**Day 0**	12.58 (2.37) 12.43 (7.45 - 17.53) n=92	12.82 (2.61) 12.62 (8.01 - 19.87) n=80	12.61 (12.12 - 13.10)	12.80 (12.22 - 13.38)	-0.19 (-0.95 - 0.56)	2.26 (0.65) 2.27 (0.77 - 4.39) n=92	2.36 (0.80) 2.18 (1.09 - 4.78) n=80	2.27 (2.13 - 2.40)	2.37 (2.19 - 2.55)	-0.10 (-0.33 - 0.12)
**Day 3**	9.47 (1.47) 9.26 (6.07 - 14.21) n=90	9.78 (1.26) 9.62 (7.22 - 12.60) n=80	9.50 (9.19 - 9.80)	9.82 (9.53 - 10.10)	-0.32 (-0.73 - 0.10)	1.67 (0.42) 1.59 (1.01 - 3.18) n=90	1.68 (0.36) 1.63 (0.96 - 2.60) n=80	1.67 (1.58 - 1.76)	1.70 (1.61 - 1.79)	-0.03 (-0.15 - 0.09)
**Day 7**	8.48 (1.49) 8.16 (5.89 - 14.17) n=89	9.16 (1.42) 9.11 (6.57 - 12.58) n=75	8.45 (8.14 - 8.76)	9.14 (8.83 - 9.46)	-0.69 (-1.13 - -0.25)	1.65 (0.46) 1.57 (0.86 - 2.95) n=89	1.75 (0.51) 1.66 (0.99 - 3.87) n=75	1.63 (1.54 - 1.73)	1.75 (1.64 - 1.86)	-0.12 (-0.26 - 0.03)
**Day 14**	8.20 (1.57) 7.97 (4.80 - 12.71) n=85	8.77 (1.70) 8.62 (4.87 - 13.71) n=74	8.21 (7.87 - 8.54)	8.78 (8.38 - 9.17)	-0.57 (-1.08 - -0.06)	1.79 (0.54) 1.69 (0.97 - 3.17) n=85	1.94 (0.61) 1.89 (0.76 - 3.45) n=74	1.79 (1.68 - 1.90)	1.96 (1.81 - 2.10)	-0.17 (-0.35 - 0.02)
**Week 4**	8.02 (1.68) 7.85 (4.22 - 12.45) n=43	8.47 (1.48) 8.11 (5.56 - 13.29) n=32				1.85 (0.59) 1.78 (0.81 - 3.90) n=43	2.14 (0.52) 2.04 (1.23 - 3.09) n=32			
**Week 6**	7.92 (0.91) 8.42 (6.79 - 8.68) n=5	8.20 (1.53) 7.84 (6.50 - 10.77) n=8				1.96 (0.21) 1.93 (1.82 - 2.32) n=5	2.00 (0.54) 1.97 (1.36 - 3.01) n=8			

© 2021 Hellström A et al. *JAMA Pediatrics*.

	AA (mol%)					DHA (mol%)				
	Raw data		MMRM			Raw data		MMRM		
Time point	Actual group: Control Mean (SD) Median (Min-Max) n	Actual group: AA:DHA Mean (SD) Median (Min-Max) n	Actual group: Control LS means (95% CI)	Actual group: AA:DHA LS means (95% CI)	Difference (AA:DHA - Control) in LS means (95% CI) p-value	Actual group: Control Mean (SD) Median (Min-Max) n	Actual group: AA:DHA Mean (SD) Median (Min-Max) n	Actual group: Control LS means (95% CI)	Actual group: AA:DHA LS means (95% CI)	Difference (AA:DHA - Control) in LS means (95% CI) p-value
**PMA 30 weeks**	7.65 (1.50) 7.64 (3.27 - 10.66) n=74	8.19 (1.66) 8.07 (5.00 - 12.52) n=64	7.70 (7.34 - 8.05)	8.15 (7.75 - 8.55)	-0.45 (-0.98 - 0.08)	2.10 (0.58) 2.00 (0.92 - 4.14) n=74	2.26 (0.58) 2.10 (1.39 - 3.89) n=64	2.07 (1.93 - 2.20)	2.25 (2.11 - 2.39)	-0.18 (-0.37 - 0.01)
**PMA 32 weeks**	7.11 (1.64) 7.15 (1.63 - 10.74) n=83	7.81 (1.99) 7.87 (2.93 - 12.64) n=72	7.10 (6.75 - 7.45)	7.81 (7.36 - 8.27)	-0.71 (-1.28 - -0.14)	2.20 (0.66) 2.27 (0.68 - 4.47) n=83	2.36 (0.68) 2.28 (0.98 - 4.28) n=72	2.15 (2.01 - 2.29)	2.38 (2.22 - 2.53)	-0.22 (-0.43 - -0.02)
**PMA 34 weeks**	6.97 (1.62) 7.08 (2.29 - 11.60) n=77	8.22 (2.34) 8.40 (3.23 - 13.70) n=69	6.77 (6.41 - 7.13)	8.25 (7.70 - 8.80)	-1.48 (-2.13 - -0.83)	2.24 (0.64) 2.29 (0.93 - 3.69) n=77	2.49 (0.80) 2.52 (0.72 - 4.30) n=69	2.20 (2.07 - 2.34)	2.51 (2.32 - 2.70)	-0.31 (-0.54 - -0.08)
**PMA 36 weeks**	7.08 (1.71) 6.98 (2.61 - 11.60) n=77	8.80 (2.15) 8.74 (4.55 - 13.91) n=64	6.94 (6.58 - 7.30)	8.75 (8.26 - 9.24)	-1.81 (-2.42 - -1.21)	2.32 (0.62) 2.35 (0.80 - 3.99) n=77	2.65 (0.79) 2.64 (0.81 - 4.42) n=64	2.28 (2.15 - 2.42)	2.63 (2.44 - 2.82)	-0.35 (-0.58 - -0.12)
**PMA 40 weeks**	6.84 (1.50) 6.99 (3.60 - 11.05) n=65	8.11 (1.88) 7.76 (4.88 - 13.52) n=65	6.87 (6.51 - 7.22)	8.11 (7.65 - 8.58)	-1.25 (-1.83 - -0.67)	2.75 (0.78) 2.79 (1.36 - 4.60) n=65	3.02 (0.78) 3.01 (1.01 - 4.69) n=65	2.69 (2.51 - 2.87)	3.04 (2.85 - 3.22)	-0.35 (-0.60 - -0.09)

Analyses are performed using Mixed Models for Repeated Measures (MMRM) data. Overall analyses were including treatment group and time points as fixed effects, and by time point analyses additionally including also an interaction term between treatment group and time points.

© 2021 Hellström A et al. *JAMA Pediatrics*.

eTable 8. Summary of Adverse Events (Safety Population)

	**Actual Group: Control (n=106)**	**Actual Group: AA:DHA (n=101)**
**Any AE (all, incl endpoints)**	106 (100.0%)	100 (99.0%)
**Any AE (prespecified, excl endpoints)**	105 (99.1%)	99 (98.0%)
**Any SAE (all, incl endpoints)**	26 (24.5%)	26 (25.7%)
**Any SAE (prespecified, excl endpoints)**	26 (24.5%)	26 (25.7%)
**Any moderate/severe AE (all, incl endpoints)**	103 (97.2%)	97 (96.0%)
**Any moderate/severe AE (prespecified, excl endpoints)**	102 (96.2%)	96 (95.0%)
**Any treatment related AE**	0 (0.0%)	1 (1.0%)
**Any AE leading to medication interruption/stop**	0 (0.0%)	13 (12.9%)
**Any AE leading to death**	13 (12.3%)	16 (15.8%)

For categorical variables n (%) is presented.
For comparison between groups Fisher´s Exact test (lowest 1-sided p-value multiplied by 2) was used for dichotomous variables.
All AEs means all reported AEs in the AE dataset, also the not pre-specified ones. Pre-specified AEs are defined a priori. All endpoints as AE means any ROP stage, any BPD, any IVH grade >1 (that were collected outside AE dataset).

© 2021 Hellström A et al. *JAMA Pediatrics*.

eTable 9. Adverse Events Including Pre-specified Terms and Studied Endpoints by Event and Category (Safety Population)

Category<br>Event	Actual Group: Control (n=106)		Actual Group: AA:DHA (n=101)	
	Events	Subjects with Events n (%)	Events	Subjects with Events n (%)
**Any AE**	**940**	**106 (100.0%)**	**818**	**100 (99.0%)**
**Circulatory**	**104**	**67 (63.2%)**	**96**	**68 (67.3%)**
Bleeding disorder	7	7 (6.6%)	6	6 (5.9%)
Bradycardia	5	5 (4.7%)	3	3 (3.0%)
Circulatory arrest	6	5 (4.7%)	7	7 (6.9%)
Circulatory instable			1	1 (1.0%)
Hypertension	1	1 (0.9%)		
Hypotension	22	16 (15.1%)	20	18 (17.8%)
Kidney failure	2	2 (1.9%)	3	3 (3.0%)
Oedema	5	4 (3.8%)	3	2 (2.0%)
PDA	51	51 (48.1%)	53	53 (52.5%)
Tachycardia	2	2 (1.9%)		
Thrombosis	3	3 (2.8%)		
**Gastrointestinal**	**42**	**23 (21.7%)**	**33**	**18 (17.8%)**
Bowel perforation	4	4 (3.8%)	7	6 (5.9%)
Cholestasis	10	10 (9.4%)	7	5 (5.0%)
Ileus	4	3 (2.8%)	3	1 (1.0%)
NEC	16	11 (10.4%)	11	10 (9.9%)
Short bowel syndrome	1	1 (0.9%)		
Suspected NEC	6	6 (5.7%)	4	4 (4.0%)
Ulcus	1	1 (0.9%)	1	1 (1.0%)
**Infection**	**129**	**65 (61.3%)**	**96**	**57 (56.4%)**
Sepsis (clinical symptoms, CRP>20 or IL-6>1000 and positive blood culture) / Suspected sepsis (clinical symptoms, CRP>20 or IL-6>1000 and negative blood culture)	89	53 (50.0%)	53	42 (41.6%)
Pneumonia	1	1 (0.9%)	5	4 (4.0%)
Other infection	39	28 (26.4%)	38	29 (28.7%)
**Metabolic**	**189**	**91 (85.8%)**	**182**	**89 (88.1%)**
Hyperbilirubinemia	81	72 (67.9%)	73	71 (70.3%)
Hyperglycemia	80	58 (54.7%)	77	56 (55.4%)

© 2021 Hellström A et al. *JAMA Pediatrics*.

Category<br>Event	Actual Group: Control (n=106)		Actual Group: AA:DHA (n=101)	
	Events	Subjects with Events n (%)	Events	Subjects with Events n (%)
Hypoglycemia	23	19 (17.9%)	29	20 (19.8%)
Nephrocalcinosis	5	5 (4.7%)	3	3 (3.0%)
**Neurological**	**43**	**32 (30.2%)**	**34**	**28 (27.7%)**
Hydrocephalus	9	8 (7.5%)	4	3 (3.0%)
IVH grade 2	14	14 (13.2%)	17	17 (16.8%)
IVH grade 3	5	5 (4.7%)	6	6 (5.9%)
IVH grade 4	12	12 (11.3%)	4	4 (4.0%)
PVL	3	3 (2.8%)	2	2 (2.0%)
Stroke			1	1 (1.0%)
**ROP**	**127**	**57 (53.8%)**	**90**	**49 (48.5%)**
ROP stage 1	27	27 (25.5%)	34	34 (33.7%)
ROP stage 2	44	44 (41.5%)	29	29 (28.7%)
ROP stage 3	33	33 (31.1%)	15	15 (14.9%)
ROP Type 1	23	23 (21.7%)	12	12 (11.9%)
**Respiratory**	**301**	**100 (94.3%)**	**279**	**96 (95.0%)**
Apnoea	52	43 (40.6%)	40	36 (35.6%)
Chronic lung disease	11	11 (10.4%)	7	7 (6.9%)
Mild BPD	39	39 (36.8%)	30	30 (29.7%)
Moderate BPD	27	27 (25.5%)	26	26 (25.7%)
Pneumothorax	3	2 (1.9%)	3	3 (3.0%)
Pulmonary Interstitial oedema	3	2 (1.9%)	1	1 (1.0%)
Pulmonary hemorrhage	6	6 (5.7%)	6	4 (4.0%)
Pulmonary hypertension	4	4 (3.8%)	8	7 (6.9%)
Pulmonary oedema	4	4 (3.8%)	4	4 (4.0%)
Respiratory insufficiency	131	83 (78.3%)	131	83 (82.2%)
Severe BPD	21	21 (19.8%)	22	22 (21.8%)
Suspected pulmonary hypertension			1	1 (1.0%)
**Other**	**5**	**5 (4.7%)**	**8**	**7 (6.9%)**
Adrenal insufficiency	1	1 (0.9%)		
Congenital anemia	2	2 (1.9%)	2	2 (2.0%)
Hypothyreosis			2	2 (2.0%)
Kidney concrement			1	1 (1.0%)
Major congenital malformation	1	1 (0.9%)		

© 2021 Hellström A et al. *JAMA Pediatrics*.

Category<br>Event	Actual Group: Control (n=106)		Actual Group: AA:DHA (n=101)	
	Events	Subjects with Events n (%)	Events	Subjects with Events n (%)
Metabolic	1	1 (0.9%)	2	2 (2.0%)
Necrosis of finger			1	1 (1.0%)

© 2021 Hellström A et al. *JAMA Pediatrics*.

eTable 10. Serious Adverse Events Including Pre-specified Terms and Studied Endpoints by Event and Category (Safety Population)

Category<br>Event	Actual Group: Control (n=106)		Actual Group: AA:DHA (n=101)	
	Events	Subjects with Events n (%)	Events	Subjects with Events n (%)
**Any AE**	**47**	**26 (24.5%)**	**45**	**26 (25.7%)**
**Circulatory**	**11**	**7 (6.6%)**	**10**	**9 (8.9%)**
Bradycardia	2	2 (1.9%)		
Circulatory arrest	5	4 (3.8%)	7	7 (6.9%)
Hypotension	2	2 (1.9%)	2	2 (2.0%)
Kidney failure	1	1 (0.9%)	1	1 (1.0%)
Thrombosis	1	1 (0.9%)		
**Gastrointestinal**	**20**	**12 (11.3%)**	**15**	**11 (10.9%)**
Bowel perforation	4	4 (3.8%)	5	5 (5.0%)
Cholestasis			2	1 (1.0%)
Ileus	3	3 (2.8%)		
NEC	12	7 (6.6%)	8	8 (7.9%)
Short bowel syndrome	1	1 (0.9%)		
**Infection**	**3**	**3 (2.8%)**	**4**	**4 (4.0%)**
Sepsis (clinical symptoms, CRP>20 or IL-6>1000 and positive blood culture) / Suspected sepsis (clinical symptoms, CRP>20 or IL-6>1000 and negative blood culture)	2	2 (1.9%)	3	3 (3.0%)
Pneumonia	1	1 (0.9%)		
Other infection			1	1 (1.0%)
**Neurological**	**2**	**1 (0.9%)**	**1**	**1 (1.0%)**
Hydrocephalus	2	1 (0.9%)		
PVL			1	1 (1.0%)
**Respiratory**	**11**	**10 (9.4%)**	**14**	**9 (8.9%)**
Chronic lung disease			2	2 (2.0%)
Pneumothorax			1	1 (1.0%)
Pulmonary hemorrhage	3	3 (2.8%)	3	3 (3.0%)
Pulmonary hypertension	4	4 (3.8%)	1	1 (1.0%)
Respiratory insufficiency	4	4 (3.8%)	7	6 (5.9%)
**Other**			**1**	**1 (1.0%)**
Necrosis of finger			1	1 (1.0%)

© 2021 Hellström A et al. *JAMA Pediatrics*.

eTable 11. Individual Causes of Death (n=29) and Doses Taken

Randomized Treatment Group	GA at birth	Cause of Death	Formulaid AA:DHA Doses Taken	Age at Death (Days)
**AA:DHA**	22+2	Renal insufficiency	2	4
**AA:DHA**	23+0	Respiratory failure	27	27
**AA:DHA**	23+5	Organ failure and metabolic acidosis	9	12
**AA:DHA**	23+6	Sepsis	3	3
**AA:DHA**	24+1	Cystic brain lesions	None	1
**AA:DHA**	24+2	Respiratory failure	34	43
**AA:DHA**	24+3	Sepsis	10	12
**AA:DHA**	24+3	NEC	11	29
**AA:DHA**	24+4	Respiratory failure and sepsis	51	69
**AA:DHA**	24+6	IVH grade 4 and circulatory collapse	1	2
**AA:DHA**	24+6	Cardiovascular collapse	2	2
**AA:DHA**	25+0	NEC, sepsis	6	7
**AA:DHA**	25+1	Cardiovascular collapse, NEC	6	7
**AA:DHA**	25+2	Respiratory failure and PVL	9	16
**AA:DHA**	25+5	Pulmonary hemmorrhage	1	3
**AA:DHA**	27+1	Cardiovascular collapse	30	34
**AA:DHA**	27+2	NEC, sepsis	21	46
**Control**	23+1	Cardiorespiratory collapse		1
**Control**	23+3	Respiratory failure		115
**Control**	24+3	Respiratory failure		39
**Control**	24+3	CMV sepsis		65
**Control**	24+3	Respiratory failure		0
**Control**	24+4	Respiratory failure		21
**Control**	24+5	IVH grade 4		2
**Control**	24+5	IVH grade 4		2
**Control**	24+5	Bradycardia		29
**Control**	25+0	Cardiorespiratory collapse		24
**Control**	25+1	IVH grade 4		2
**Control**	26+2	Sepsis, cerebral abscess, cardiac thrombosis		39

© 2021 Hellström A et al. *JAMA Pediatrics*.

eReferences.

1. Walsh MC, Kliegman RM. Necrotizing enterocolitis: treatment based on staging criteria. Pediatr Clin North Am 1986;33:179-201.
2. Niklasson A, Albertsson-Wikland K. Continuous growth reference from 24th week of gestation to 24 months by gender. BMC Pediatr 2008;8:8.

© 2021 Hellström A et al. *JAMA Pediatrics*.
