# Supplementary material for: Effect of Enteral Lipid Supplement on Severe Retinopathy of Prematurity: A Randomized Clinical Trial
Source: JAMA Pediatr. 2021 Feb 1;175(4):1–9. doi: 10.1001/jamapediatrics.2020.5653 (PMC7851754; doi:10.1001/jamapediatrics.2020.5653)
Supplement: Supplement 3. — Data Sharing Statement [file jamapediatr-e205653-s003.pdf]

Data Sharing Statement

Hellström. Effect of Enteral Lipid Supplement on Severe Retinopathy of Prematurity. *JAMA Pediatr*. Published February 01, 2021. doi:10.1001/jamapediatrics.2020.5653

Data
Data available: No

Additional Information
Explanation for why data not available: According to GDPR and PDL we have no legal approval to share individual data, if there is interest of a research collaboration a material transfer agreement and amendment for ethical approval has to be conducted.
